# Metabolic Radiosensitization by Targeting Lactate Metabolism with Microfluidic Liposomal Nanocarriers

**DOI:** 10.1021/acsbiomaterials.5c02175

**Published:** 2026-01-28

**Authors:** Meabh Doherty, Jie Feng, Tongchuan Wang, Cancan Yin, Niall M. Byrne, Sarah Chambers, Rayhanul Islam, Dimitrios A. Lamprou, Jonathan A. Coulter

**Affiliations:** School of Pharmacy, Queens University Belfast, Lisburn Road, Belfast BT9 7BL, U.K.

**Keywords:** lactate metabolism, MCT1/4, MPC, hypoxia

## Abstract

Lactate, the main product of the Warburg effect, exerts both intrinsic effects on cancer cell metabolism and noncell autonomous effects that promote tumor development, metastasis, and treatment resistance. As such, glycolytic dependence in tumors is frequently associated with poor clinical outcomes. Targeting lactate metabolism has emerged as a promising strategy to enhance the efficacy of conventional therapies. Here, we investigate the therapeutic potential of targeting lactate metabolism via inhibiting MCT1, MCT4, and MPC in PC3 and FaDu tumor cell models. We confirmed lactate as a substrate that fuels mitochondrial respiration and supports cell survival under hypoxic conditions. Inhibition of lactate influx mediated by 7ACC2 reduced oxygen consumption, sensitizing tumor cells to radiation in both 2D-cell cultures and 3D-spheroid models. Encapsulation of 7ACC2 in DPPC liposomes using microfluidics preserved radiosensitizing activity in both systems, promoting reoxygenation, while overcoming the pharmacological limitations of the free drug. This liposomal formulation therefore represents a promising therapeutic approach to help mitigate hypoxia-induced radioresistance.

## Graphical abstract

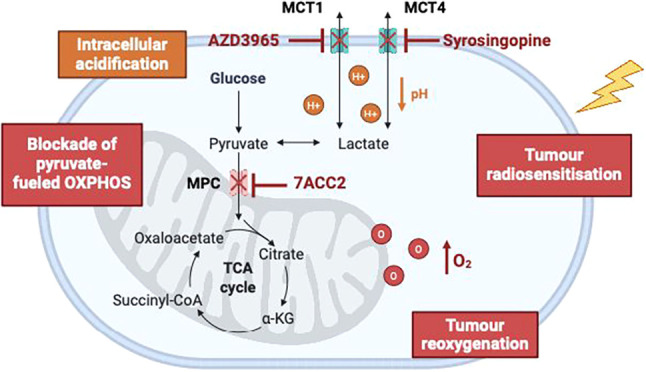

## 1 Introduction

Metabolic reprogramming, a recognized cancer hallmark, enables tumor cells to support rapid growth, adapting to the stresses of a diverse and often unfavorable microenvironment.^1^ Altered utilization and transport of lactate, a byproduct of glycolysis, is reported in various solid tumors, including head-and-neck cancer and prostate cancer.^2,3^ The impact of lactate metabolism is correlated with various downstream pro-tumor responses, including the establishment of an immunosuppressive tumor microenvironment and increased oxidative scavenging, contributing to chemo- and radioresistance.^4^ Thus, altered lactate metabolism has emerged as an alternative therapeutic strategy that could enhance the effectiveness of conventional cancer treatments.

Within the complex cellular machinery involved in lactate metabolism, several promising targets, including lactate dehydrogenase (LDH), monocarboxylate transporter 1 (MCT1), monocarboxylate transporter 4 (MCT4), and mitochondrial pyruvate carrier (MPC), have been implicated in modulating tumorigenesis.^5^ MCTs are a diverse group of transmembrane proteins encoded by SLC16 genes, playing a critical role in regulating intracellular and extracellular lactate levels.^6,7^ Among them, MCT1 and MCT4 are particularly important for tissue metabolic activity through proton-coupled transport of monocarboxylates such as l-lactate, ketone bodies, and pyruvate.^8^ MCT1 exhibits a higher affinity for lactate, is ubiquitously expressed, and primarily mediates lactate import along with other monocarboxylates.^9,10^

In contrast, MCT4 has a lower lactate affinity and is predominantly found in lactate-producing, glycolysis-dependent cells, regulated by hypoxia through HIF-1α-dependent mechanisms that enable the efflux of intracellular lactate.^11,12^ Overexpression of both MCT1 and MCT4 is reported in various cancer types and are associated with higher TNM staging and poorer patient outcomes.^13–15^ As such, several studies have investigated the potential of MCTs as a therapeutic target, particularly in relation to sensitizing to DNA damaging agents.^16,17^ Saulle et al. (2021) found that blocking lactate transport using AR-C15585818 and syrosingopine, MCT1 and MCT4 inhibitors, respectively, impaired leukemia cell proliferation, increasing sensitivity to the chemotherapeutic arabinosylcytosine.^18^ Similar radiosensitizing effects were reported when an MCT1 inhibitor was combined with α-cyano-4-hydroxycinnamate (CHC) and radiation, selectively killing hypoxic/glycolytic tumor cells.^19^ Furthermore, selective MCT1 inhibition using AZD3965 yielded significant antitumor effects when combined with radiation, increasing intracellular acidification through lactate retention, suppressing tumor growth, and improving survival.^20,21^ However, MCT4 overexpression can confer resistance to MCT1 inhibition, outlining the need to consider dual-target strategies.^21,22^

MPC is formed by two proteins encoded by the genes *MPC1* and *MPC2*, which belong to the SLC54 family of mitochondrial transporters.^23^ MPCs are located on the inner mitochondrial membrane and act as critical regulators of glycolysis, mitochondrial respiration, and lactate production, all of which are associated with increased tumorigenesis.^23–25^ Consequently, targeting the mitochondrial pyruvate carrier has emerged as a potential therapeutic strategy in preclinical models.

Pharmacological inhibition of MPC using 7-aminocarboxycoumarin (7ACC2) markedly reduces lactate influx and suppresses tumor growth across various *in vitro* tumor models, including cervical, breast, head and neck, and pancreatic cancers.^26–28^ Corbet et al. showed that 7ACC2 blocked MPC function, inhibiting lactate influx and inducing cytostatic effects, resulting in reduced spheroid hypoxia, a response likely to increase radiation sensitivity due to the radiosensitizing effects of oxygen.^26^ In a SiHa cervical cancer xenograft model, combining 7-aminocarboxycoumarin with radiation substantially enhanced antitumor efficacy, delaying tumor growth. These findings implicate MPC as a promising target for improving radiotherapy outcomes alone or in combination with conventional treatments.

Despite encouraging preclinical results, inhibitors of MCTs or MPC face barriers that limit clinical utility, including poor solubility, low bioavailability, rapid metabolism, and ineffective cellular transport.^29,30^ Liposomal delivery presents a solution to these pharmacodynamic barriers, providing a lipophilic bilayer, encapsulating the drug within an aqueous core.^31^ This architecture enables the encapsulation of hydrophobic compounds, such as 7ACC2, enhancing both stability and bioavailability. Conventional bulk liposome preparation methods suffer from limited reproducibility with poor control over particle size and polydispersity, resulting in scale-up challenges.^32,33^ In contrast, microfluidic technology overcomes these limitations, providing precise control over liposome diameter, surface properties, and encapsulation efficiency while improving batch uniformity.^34,35^

Building on this background, this study targets lactate metabolism through MCT1, MCT4, and MPC inhibition. We focus on the radiosensitizing potential of MPC inhibition to overcome hypoxia-induced radioresistance, outlining a microfluidics-based liposomal formulation of 7ACC2, optimizing drug delivery while maintaining radiosensitizing efficacy in tumor models of prostate and head-and-neck cancer.

## 2 Materials and Methods

### 2.1 Chemicals and Reagents

AZD3965 and 7ACC2 (7-aminocarboxycoumarin 2) were purchased from MedChem Express. Syrosingopine, 1,2-dipalmitoyl-*sn*-glycero-3-phosphocholine (DPPC), and cholesterol were obtained from Sigma-Aldrich. Stock solutions of AZD3965 (5 mM), syrosingopine (10 mM), and 7ACC2 (50 mM) were prepared in 100% dimethyl sulfoxide (DMSO) and stored at −20 °C. Primary antibodies against α-tubulin (No. 2144S) and MPC1 (No. 14462S) were obtained from Cell Signaling Technology. Antibodies against MCT1 (ab9308), MCT4 (ab234728), and HRP-conjugated goat antirabbit IgG (ab205718) were purchased from Abcam.

### 2.2 Cell Culture

FaDu (HPV(−)­HNSCC) and PC3 (prostate cancer) cell lines were sourced from the American Type Culture Collection (ATCC) and routinely tested for mycoplasma contamination (Lonza). FaDu cells were maintained in Eagle’s Minimum Essential Medium (EMEM), and PC3 cells were maintained in RPMI-1640 (Gibco), supplemented with 10% fetal bovine serum (FBS, Gibco). Cells were cultured at 37 °C in a humidified 5% CO_2_ atmosphere. For hypoxia experiments, cells were incubated at 0.5% O_2_ in an InvivO^2^ hypoxic chamber (Baker, Bridgend, U.K.).

### 2.3 Western Blotting

Cells were washed with cold PBS and lysed in a RIPA buffer (Pierce) containing 1% protease and phosphatase inhibitors (Thermo Fisher). Protein concentrations were quantified using a NanoDrop 2000c. Equal amounts of protein (30 μg) were separated by 4–12% SDS-PAGE electrophoresis (Invitrogen) and then transferred to nitrocellulose membranes (Amersham Protran). Membranes were blocked with 5% semiskimmed milk in PBST (0.1% Tween-20 in PBS) for 1 h at room temperature, followed by overnight incubation at 4 °C with primary antibodies (1:1000 in PBST). After being washed, membranes were incubated with HRP-conjugated secondary antibody (1:2000) for 1 h at room temperature. Protein bands were visualized by chemiluminescence and imaged using a UVI Tech system (Cambridge, U.K.). Semiquantitative analysis was performed in ImageJ, normalizing protein expression to GAPDH or α-tubulin.

### 2.4 Radiation Clonogenic Assay

To assess the radiosensitizing effects of MCT or MPC inhibition, 2 × 10^5^ cells were seeded in six-well plates and allowed to adhere overnight. Cells were then treated for 24 h with 1 μM AZD3965, 10 μM syrosingopine, 10 μM 7ACC2, or 7ACC2-loaded liposomes prior to irradiation with 160 kVp X-rays. For hypoxic experiments, cells were incubated at 0.5% O_2_ for 4 h, then treated with the same inhibitors for a further 24 h under hypoxia before irradiation in airtight bags. After irradiation, cells were washed with PBS, detached, counted, and reseeded at low density under normoxia for colony formation over 9–14 days. Colonies were fixed and stained with 0.4% crystal violet in 70% methanol. Colonies containing >50 cells were counted to calculate plating efficiency (PE). Surviving fraction (SF) was determined relative to nonirradiated controls and fitted to the linear-quadratic (LQ) model (eq 1) to derive radiobiological parameters, including the α/β ratio, sensitizer enhancement ratio (SER), and dose enhancement factor (DEF).

1 $$S\left(D\right)=\exp\left(-\alpha D-{\beta D}^{2}\right)$$

### 2.5 Spheroid Growth Delay Assay (SGD)

FaDu cells were seeded at 1 × 10^3^ cells per well in 200 μL of complete media using ultralow attachment 96-well U-bottom plates. Spheroids were left to form over 3 days, with 8 technical replicates per treatment group and three independent replicates. Once formed, 100 μL of medium was replaced with fresh medium containing either 7ACC2 (10 μM), liposomal 7ACC2 (100 μM loaded), or 0.1% DMSO as a vehicle control. After 24 h, spheroids were irradiated with a single dose of 6 or 9 Gy. Growth was monitored over 2 weeks using the Cell3iMager system (SCREEN, Japan), with media replenished by 50% volume exchange.

Spheroids were costained with a hypoxia live cell marker (Image-iT, Thermo Fisher) and Hoechst dye (Invitrogen) when reaching a diameter of ∼600 μm. Co-staining involved replacing 100 μL of medium with dye-containing medium (5 μM hypoxia dye and 10 μg/mL Hoechst), followed by a 1–2 h incubation at 37 °C, protected from light exposure. Fluorescence imaging was performed using an Agilent BioTek Cytation 5 plate reader, and hypoxia signals were quantified using Gen5 software.

### 2.6 Succinate Assay

PC3 and FaDu cells were seeded at 1 × 10^6^ cells in a P60 dish and incubated overnight, allowing attachment. Media were then replaced with fresh media containing either 7ACC2 or liposomal 7ACC2 for 24 h. Cells were washed with cold PBS, trypsinized, and neutralized with 0.5 mL complete medium. After centrifugation (400 g, 5 min), cells were counted, recentrifuged, and homogenized in cold (4 °C) succinate assay buffer (SAB, Abcam ab204718).

Lysates were centrifuged (15,000*g*, 5 min, 4 °C), filtered through a 10 kDa spin column (Abcam ab93349), and then diluted in 120 μL in SAB. Samples (50 μL) were incubated with reaction buffer at 37 °C for 30 min in the dark, and absorbance was measured at 450 nm. Intracellular succinate levels were quantified using a standard curve and normalized to untreated controls.

### 2.7 Seahorse XFe Metabolic Flux Analysis

Cellular bioenergetics of PC3 and FaDu cells treated with 7ACC2 were assessed using a Seahorse XFe96 Analyzer (Seahorse Bioscience, MA, USA). Cells were seeded at 2 × 10^4^ cells/well and incubated overnight. Prior to the assaying, cells were washed with Seahorse XF assay media (10 mM glucose, 1 mM sodium pyruvate, 2 mM l-glutamine, pH 7.4) and then incubated in 180 μL of Seahorse assay media in a non-CO_2_ incubator for 1 h. A 20 μL solution of 7ACC2 (100 μM) was loaded into the injection port of the XFe96 sensor cartridge. After establishing a baseline oxygen consumption rate (OCR) and extracellular acidification rate (ECAR), 7ACC2 was automatically injected. Changes in OCR and ECAR were recorded over 3 h and expressed as a percentage of baseline values.

### 2.8 Preparation of Liposomal 7ACC2 via Microfluidics

Liposomal encapsulation of 7ACC2 was achieved using a Dolomite microfluidics system (Figure S1) with a Y-shaped micromixer chip (52 mm × 36 mm, channels: 300 μm width, 130 μm height, staggered herringbone design). The lipid phase (DPPC: cholesterol, 2:1) was dissolved in ethanol at 1 mg/mL and mixed with 7ACC2 (10–1000 μM), followed by sonication until fully solubilized. The aqueous phase consisted of PBS (pH 7.4).

Using pressure pumps, the lipid and aqueous phases were injected into separate inlets of the microfluidics chip. Total flow rate (TFR) and flow rate ratio (FRR) were monitored using mitos flow rate sensors (0.2–5 mL/min). Optimal conditions were TFR = 4 mL/min and FRR = 1:3 (lipid: aqueous), resulting in efficient liposome formation.

### 2.9 Characterization of Lipo-7ACC2 (Particle Size, ζ-Potential, Atomic Force Microscopy)

Particle size and polydispersity index were determined using dynamic light scattering (DLS) using a Nano brook Omni DLS instrument (Brookhaven Instruments, Holtsville, NY, USA). For size and charge analysis, lipo-7ACC2 was diluted in PBS (1:100) for analysis. Particle measurements were then collected three times at 20 °C at a fixed angle of 90°. The ζ-potential was measured using the same instrument through phase analysis light scattering (PALS). Liposome morphology (empty and encapsulated) was also examined using atomic force microscopy (AFM) TT-2 instrument (AFM Workshop, US). Each sample (20 μL) was diluted with PBS to a final volume of 2 mL. From this solution, 20 μL was deposited onto freshly cleaved mica substrates (1.5 cm × 1.5 cm; G250–2 mica sheets, 1 in. × 1 in. × 0.006 in.; Agar Scientific Ltd., Essex, U.K.) and allowed to air-dry for 30 min. Substrates were then gently rinsed with 1 mL PBS to remove nonadherent liposomes, followed by an additional 30 min drying period. AFM imaging was then performed using Ohm-cm antimony-doped silicon probes with resonance frequencies of 50–100 kHz. Images were acquired at a resolution of 512 × 512 pixels and a scan rate of 0.6 Hz.

### 2.10 *In Vitro* Release Study and Encapsulation Efficiency

The release profile of 7ACC2 was evaluated by using a dynamic dialysis method. Dialysis tubing (cellulose membrane, 10 mm flat width, MWCO 14,000; Sigma-Aldrich) was sterilized in boiling water for 30 min and then rinsed with deionized water. 1 mL of liposome suspension was placed in the dialysis bag and then sealed at both ends. Due to the low solubility of 7ACC2 in aqueous media, release buffer (PBS) was supplemented with either 2% Tween 80 or lysozyme (3 mg/mL), simulating physiological conditions. Dialysis tubes were submerged in 7 mL of release buffer and incubated at 37 °C. Samples were collected at 1 h, 1 day, and three times weekly for up to 3 weeks. At each time point, 1 mL of the supernatant was withdrawn and replaced with prewarmed, equilibrated buffer, maintaining sink conditions.

Released 7ACC2 was quantified using a UV–vis spectrophotometer (Jenway 7205) at 418 nm, with calibration based on a standard curve in ethanol (*R* ^2^ = 0.9952). Concentration was calculated by using eq 2

2 $$\mathrm{concentration}\left(\mu M\right)=\frac{\mathrm{absorbance}-0.0377}{0.0104}$$

Encapsulation efficiency (EE) of 7ACC2 was calculated using eq 3

3 $$\mathrm{EE}\left(\%\right)=\frac{C_{i}-C_{f}}{C_{i}}\times 100$$

where *C_i_* is the initial concentration used in liposome preparation and *C*_f_ is the concentration of free, nonencapsulated drug.

Cumulative release was plotted as a function of time using eq 4

4 $$cumulative\;curcu\min\;release\left(\%\right)=\frac{C_{t}}{C_{e}}\times 100$$

where *C_t_* is the concentration of 7ACC2 released at time *t* and *C*_e_ is the total amount encapsulated.

### 2.11 Statistical Analysis

All data were plotted and analyzed using GraphPad Prism 9, reported as mean ±  standard deviation (SD). For clonogenic assays, two-way ANOVA followed by Tukey’s multiple comparisons test was used. For all other assays, one-way ANOVA with Tukey’s test was applied. Statistical significance was defined as *p* < 0.05.

## 3 Results

### 3.1 MCT1/4 and MPC Expression in PC3 and FaDu Cells

To assess the baseline expression of lactate transporters and mitochondrial pyruvate carriers, we first examined MCT1/4 and MPC levels in PC3 and FaDu cells under normoxia (21% O_2_). Both cell lines expressed MCT1, MCT4, and MPC (Figure 1a). Notably, MCT1 expression was higher in FaDu cells compared to PC3 cells (2.6-fold, *p* = 0.0027; Figure 1a). We then assessed the impact of hypoxia (0.5% O_2_), given its role in promoting glycolysis and lactate production. Surprisingly, hypoxia did not significantly alter the MCT1 or MCT4 expression in PC3 cells (Figure 1b). However, MPC expression was significantly reduced under hypoxia (*p* = 0.0498), with PC3 cells showing an ∼50% decrease. In FaDu cells, hypoxia had no effect on the MCT1/4 or MPC expression (Figure 1c).

**Figure 1:**
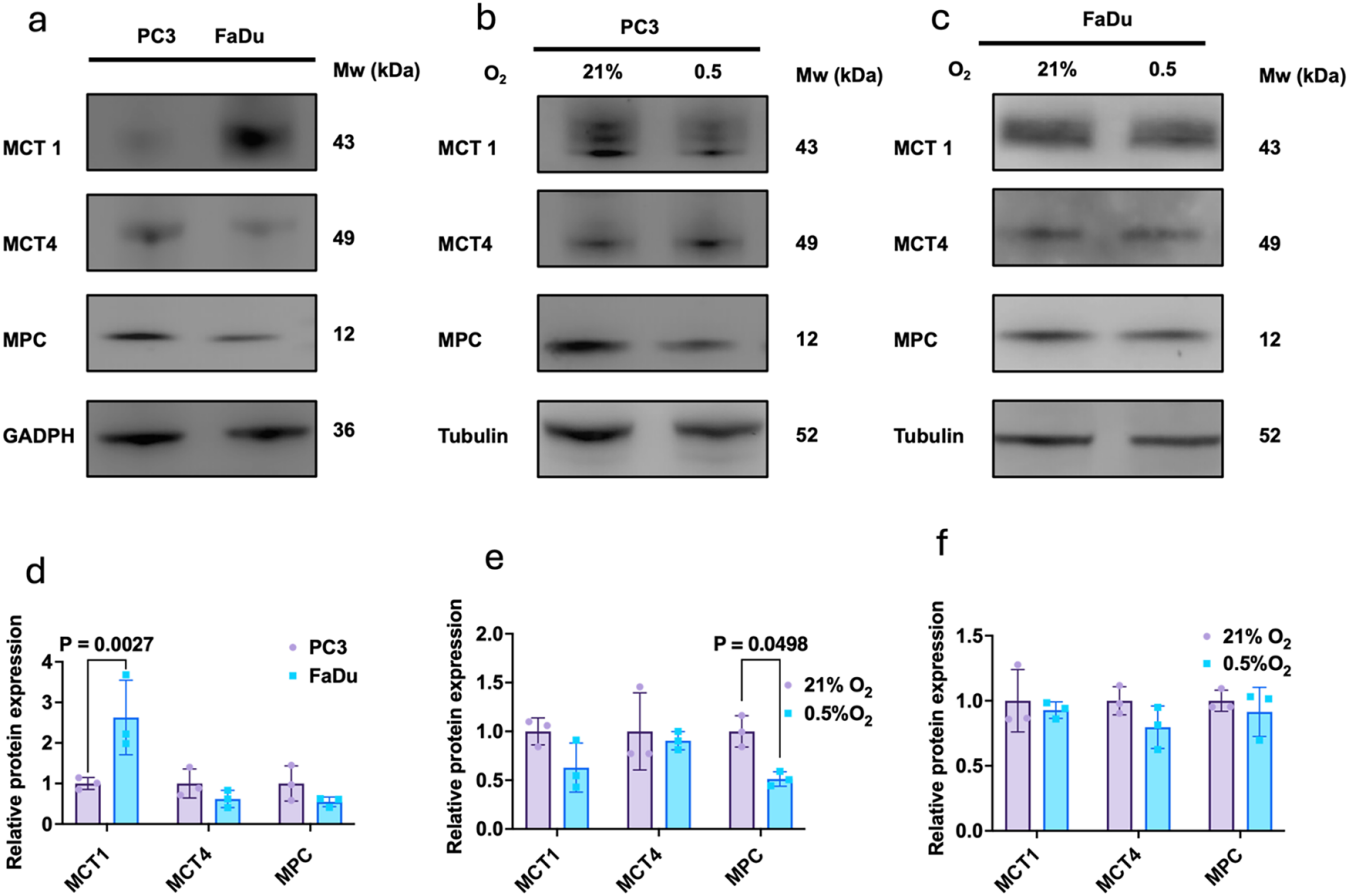
Basal expression of MCT and MPC in PC3 and FaDu cells. Representative immunoblots (a) and densitometric quantification (d) of basal MCT1, MCT4, and MPC expressions in PC3 and FaDu cells. Representative immunoblots (b) and quantification (e) show MCT1, MCT4, and MPC expression in PC3 cells under normoxia (21% O_2_) or hypoxia (0.5% O_2_), while representative immunoblots (c) and quantification (f) show the same proteins in FaDu cells under these conditions. Data represent the mean ± SD from three independent experiments. Statistical significance was determined using a two-tailed Student’s *t* test.

### 3.2 Evaluation of Therapeutic Potential of Targeting Lactate Metabolism via MCT1/4, MPC Inhibition

We next examined how lactate or glucose availability influences the effect of AZD3965, syrosingopine, and 7ACC2 on the cell viability. Under normoxia and glucose restriction (lactate supplementation), inhibition of MCT1 or MPC alone had no significant effect on the PC3 cells. However, dual inhibition of MCT1 and MPC significantly reduced viability by 27% (*p* = 0.0218; Figure 2a). Combined MCT1, MCT4, and MPC inhibition further decreased viability by 42.4% over controls (*p =* 0.0011; Figure 4a). In glucose-rich conditions, single and combined MCT inhibition had no impact on viability, with only complete MCT blockade coupled with MPC inhibition inducing a significant 44% reduction in viability. In FaDu cells, 7ACC2-mediated inhibition of pyruvate transport alone significantly (*p* < 0.0001) reduced viability by 35.7% under conditions of lactate supplementation (Figure 2b), increasing to 55% (*p* = 0.0173) with combined MCT inhibition, an effect maintained at 52% reduction (*p* = 0.0003) under glucose-rich conditions. Under hypoxic conditions, PC3 cells proved more susceptible to individual MCT1 and MPC inhibition, where lactate was the primary fuel source, with MPC inhibition reducing survival by >50% (*p* < 0.0001; Figure 2c). Dual or triple combinations did not provide an additional benefit. Similar trends were observed in FaDu cells (Figure 2d).

**Figure 2:**
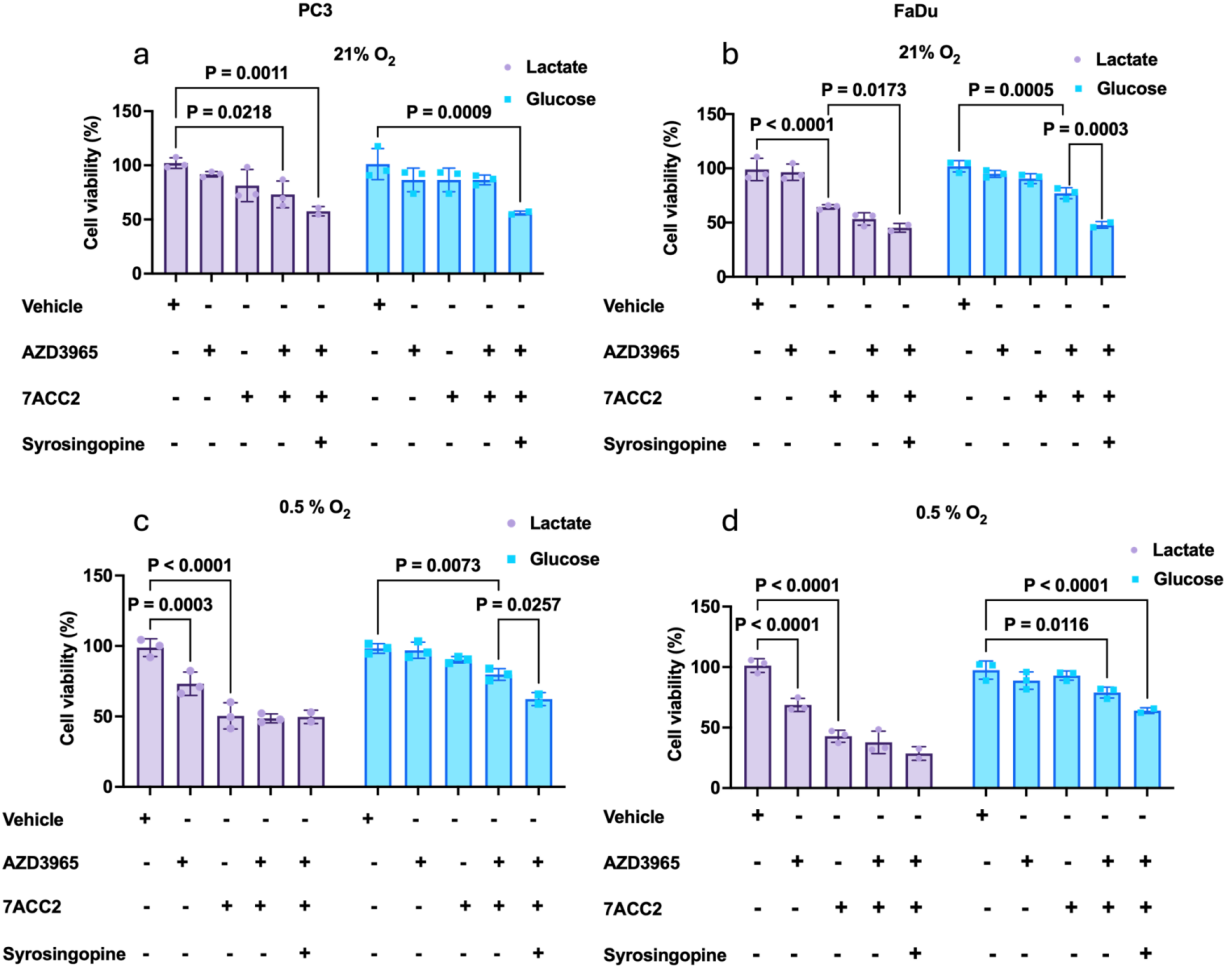
Inhibitors of lactate metabolism influence tumor cell growth depending on substrate availability (lactate or glucose). PC3 and FaDu cells were treated with AZD3965 (1 μM), syrosingopine (10 μM), 7ACC2 (10 μM), or in combination in complete media supplemented with either lactate or glucose for 72 h under normoxic (a–b) and hypoxic (c–d) conditions. Data represent mean ± SD from three independent replicates. Statistical significance was assessed using one-way ANOVA with Tukey’s posthoc test.

To investigate how interference of lactate metabolism may affect lactate transport, we next assessed the impact of MCT and MPC inhibition on the extracellular pH (pHe). Inhibition of MCT1 using AZD3965 significantly reduced acidity levels of the extracellular media in both PC3 (*p* = 0.0033; Figure S2a) and FaDu cells (*p* = 0.0225; Figure S2b), indicating reduced lactate export. In contrast, syrosingopine, which targets MCT4, did not alter the pHe. Combined MCT1/4 inhibition showed no additive effect, pointing to MCT1 as the primary mediator of lactate efflux (Figure S2a–b). MPC inhibition with 7ACC2 is expected to elevate cytosolic pyruvate, promoting lactate production and efflux and protecting against intracellular acidification. As hypothesized, MPC inhibition significantly acidified PC3 media (*p* = 0.0025; Figure S2c). Co-treatment with MCT1/4 inhibitors blocked this acidification, supporting the role of MCTs in lactate export. FaDu cells produced a similar, though less pronounced, response (Figure S2d)

### 3.3 Inhibiting Lactate Metabolism Enhances Radiation Sensitivity

Given the role of lactate symbiosis in supporting tumor cell survival, we investigated whether combined MCT and MPC inhibition could overcome hypoxia-induced radioresistance. Unsurprisingly, hypoxia significantly increased radiation resistance (Figure S3), yielding an oxygen enhancement ratio (OER) of 1.91 (PC3) and 1.81 (FaDu), confirming maintenance of hypoxia during radiation treatment (Table S2). In the absence of radiation, neither AZD3965 nor the combined treatment showed any significant toxicity, as evidenced by the minimal change in SF_0Gy_ value (Figure S4). MCT1 inhibition with AZD3965 did not significantly affect the radiation response in PC3 cells under either oxygen condition (Figure 3a–b). In contrast, MPC inhibition with 7ACC2 significantly increased radiation sensitivity under both normoxia (*p* = 0.0053) and hypoxia (*p* = 0.0004), producing SER values of 1.19 and 1.2, respectively (Figure 3a,b and Table S3). Adding MCT1 or MCT4 inhibitors did not further enhance this effect, indicating the radiosensitizing impact is primarily due to MPC inhibition. In FaDu cells, 7ACC2 also significantly increased radiosensitivity by 11% under normoxia (*p* = 0.0404; Figure 3c). However, it had no effect under hypoxia unless combined with AZD3965 and syrosingopine, which significantly improved radiosensitivity (*p* = 0.047), yielding a SER of 1.14 (Figure 3d and Table S3).

**Figure 3:**
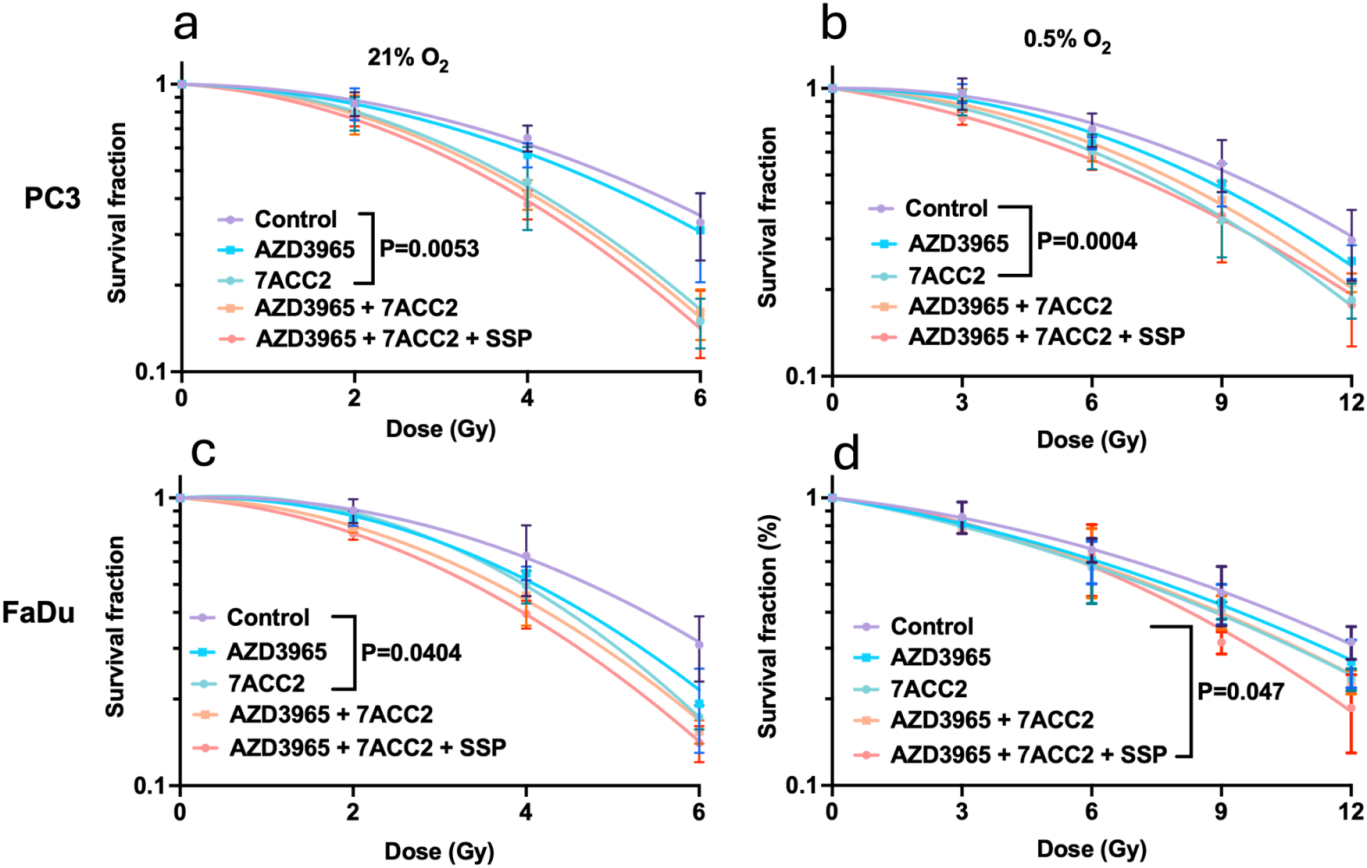
Inhibiting lactate metabolism sensitizes tumor cells to radiation under both normoxic and hypoxic conditions. Clonogenic survival curves for PC3 (a, b) and FaDu (c, d) cells treated with AZD3965 (1 μM), syrosingopine (10 μM), 7ACC2 (10 μM), or combinations for 24 h under normoxia (a, c—21% O_2_) or hypoxia (b, d—0.5% O_2_). Data represent the mean ± SD of three independent experiments. Statistical significance was assessed using two-way ANOVA with Tukey’s multiple comparison test.

### 3.4 Blocking MPC via 7ACC2 Impaired TCA Cycle Reducing Oxygen Consumption

Next, we used a Seahorse XF Analyzer to establish if 7ACC2-mediated MPC inhibition alters tumor cell bioenergetic profiles in PC3 (Figure 4a,b) and FaDu (Figure 4c,d) cells, analyzing both OCR and ECAR, reflecting mitochondrial respiration and glycolysis. 7ACC2 reduced OCR by 20 and 35% in PC3 and FaDu cells, respectively. In parallel, ECAR was significantly elevated (PC3–20%; FaDu—50%), indicating a switch toward a glycolytic dependence.

**Figure 4:**
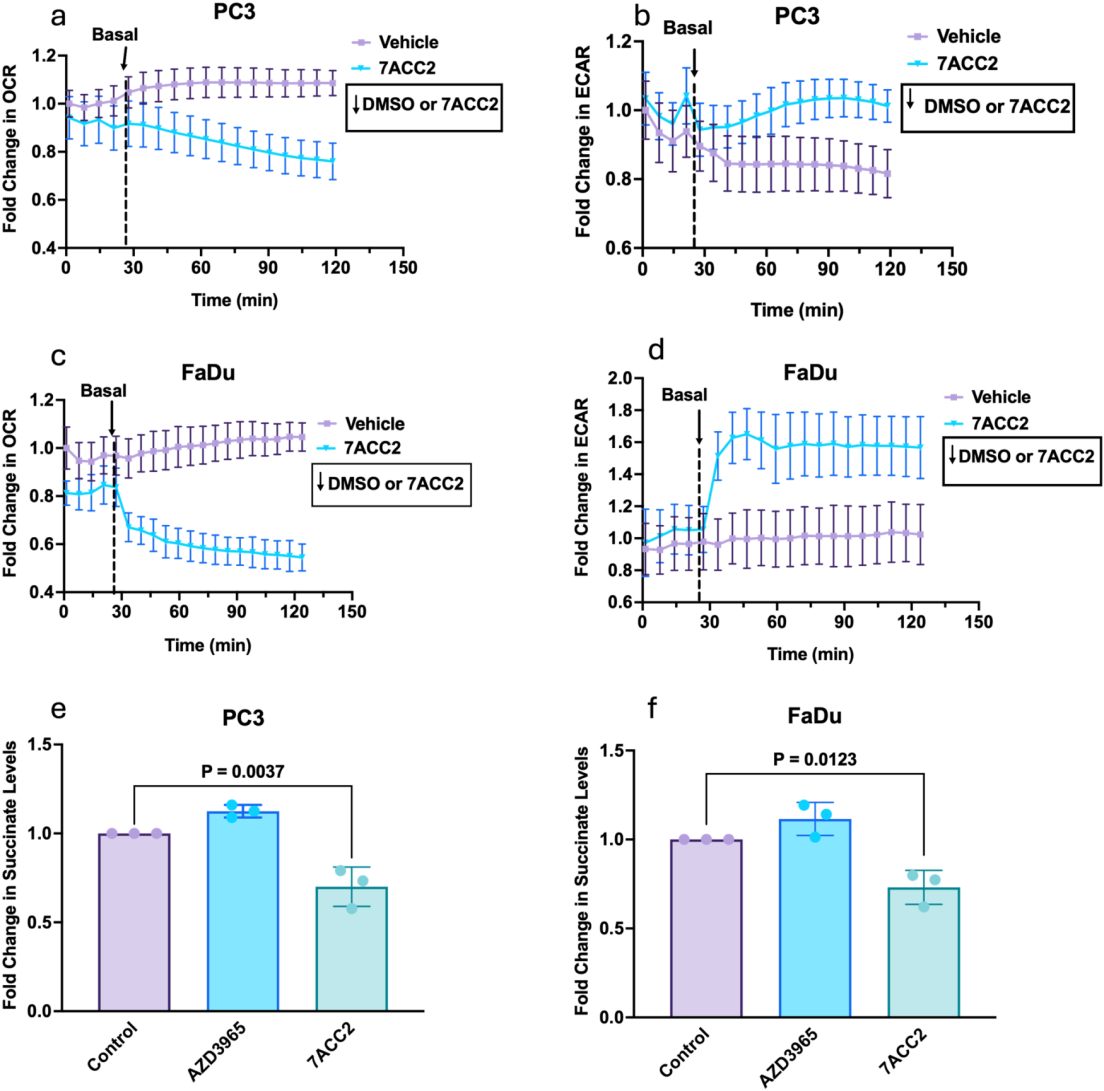
7ACC2 inhibition of MPC reduces oxygen consumption and TCA cycle activity. Bioenergetic profile of PC3 (a, b) and FaDu (c, d) cells treated with 7ACC2. Oxygen consumption rate (OCR) and extracellular acidification rate (ECAR) were monitored at 37 °C for 2 h, reported as percentage change over baseline following treatment. Data are presented as mean ± SD (*n* = 3 per treatment group). Relative levels of succinate were observed in PC3 (e) and FaDu (f) cells after 7ACC2 (10 μM) treatment for 24 h. Data derived from three independent replicates, presented as mean ± SD. Statistical differences determined by one-way ANOVA with a Tukey multiple comparison test.

Given the activity of MPC in facilitating mitochondrial pyruvate influx, fueling the TCA cycle, we also assessed the extent by which 7ACC2 suppressed succinate levels, a key intermediate of pyruvate metabolism, a regulator of HIF-1α stabilization. As anticipated, succinate levels were significantly antagonized in both PC3 (*p* = 0.0037) and FaDu cells (*p* = 0.00123) (Figure 4e–f,), by 30 and 27%, respectively.

### 3.5 MPC Blockade Affects Spheroid Oxygenation and Radiation Response

Given the potential of MPC inhibition in reducing the level of OCR and increasing 2D-cell culture radiation sensitivity, we next investigated whether MPC inhibition could modulate oxygenation levels of 3D-spheroid models. FaDu spheroids were treated with 7ACC2, stained with Image-iT green hypoxia dye, and counterstained with Hoechst. Under low-oxygen tensions, the Image-iT dye is converted to a fluorescent form, with intensity positively correlating with increasing hypoxia. Figure 7a illustrates both untreated and vehicle-only spheroids, each possessing a large necrotic core indicated by the dark central core. 7ACC significantly reduced the size of the necrotic core. Image-iT staining confirmed a significant 7ACC2-mediated reduction in green fluorescence over vehicle-only (15.95 au vs 50.08 au; Figure 5a–b), indicating increased oxygenation. Quantification of the hypoxic ratio (defined as hypoxic core diameter/spheroid diameter) revealed that 7ACC2 treatment markedly reduced hypoxic core size (*p* < 0.0001), independent of radiation dose (Figure 5c–d). Specifically, 7ACC2 alone decreased the hypoxic core by 34.6% compared with the vehicle control. A similar effect was observed when combined with radiation, where 7ACC2 further reduced the hypoxic ratio by over 42% relative to that of 7ACC2 alone. Taken together, these results suggest that the observed decrease in the level of the OCR induced by 7ACC2 corresponds to decreased spheroid hypoxia.

**Figure 5:**
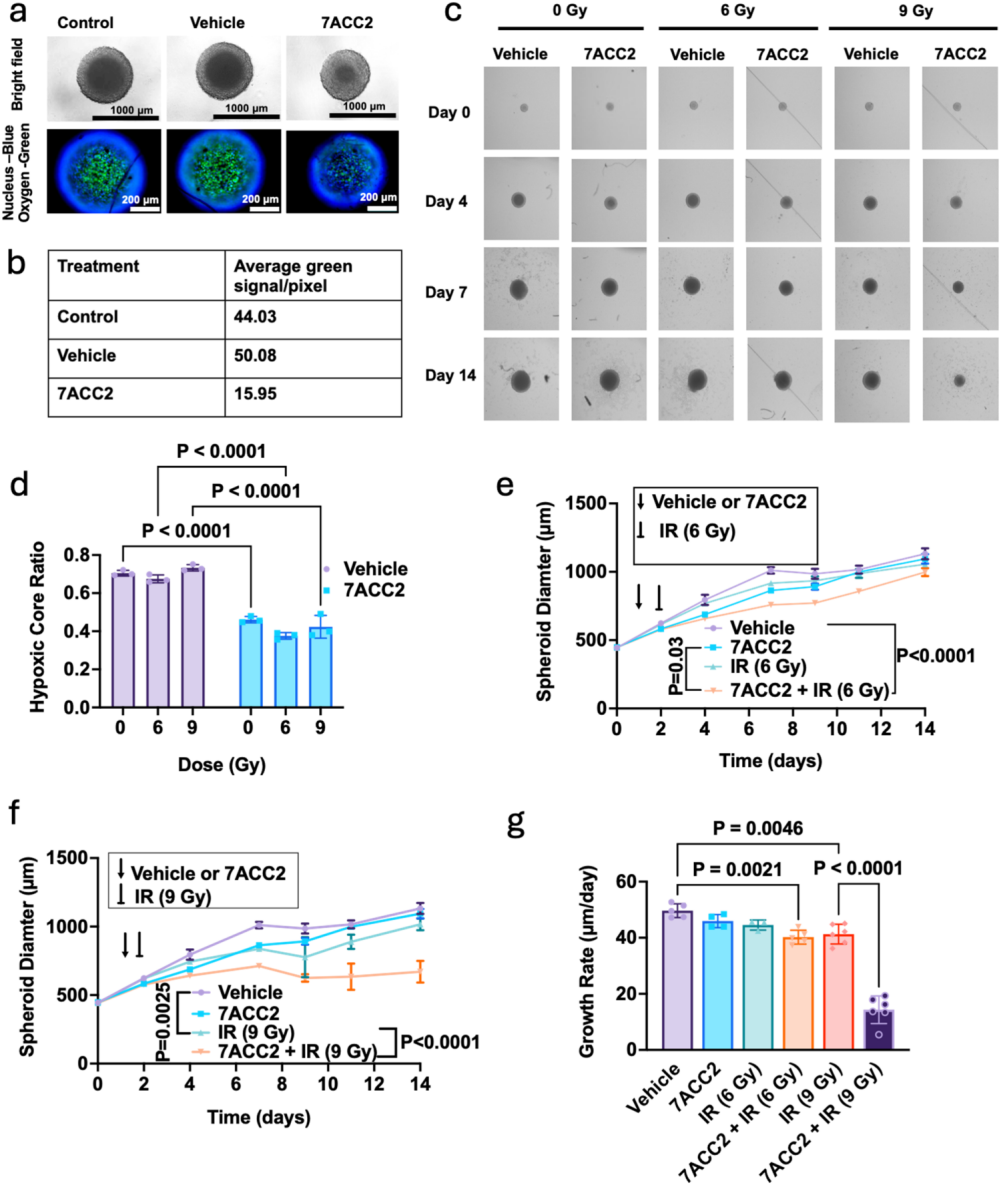
MPC inhibition alleviates spheroid hypoxia, increasing radiation sensitivity. (a) Confocal fluorescence and bright-field imaging of FaDu spheroids (∼500 μm) treated with 0.1% DMSO as vehicle or 7ACC2 (10 μM) for 24 h, followed by Image-iT green hypoxia dye and Hoechst nuclear staining. (b) Semiquantitative analysis of image-iT green fluorescence using ImageJ color histogram analysis. (c) Representative images of unirradiated or radiated FaDu spheroids treated with DMSO or 7ACC2 over 14 days post-treatment. (d) Quantitative assessment of the relative hypoxic core ratio in un- or radiated FaDu spheroids treated with DMSO or 7ACC2. (e, f) FaDu spheroid growth delay (SGD). FaDu cells (1000 cells/well) were seeded in ultralow attachment 96-well plates, forming spheroids over 3 days. Spheroids were treated with vehicle (DMSO) or 7ACC2 for 24 h prior to radiation treatment (single fraction 6 or 9 Gy; 160 kVp X-ray). Mean data derived from a minimum of *n* = 8 spheroids per treatment condition. (g) Mean daily growth rate from SGD assays defined by increased spheroid diameters per day in culture. Statistical differences were determined by one-way ANOVA with a Tukey multiple comparison test.

To assess whether hypoxia alleviation enhanced spheroid radiation sensitivity, FaDu spheroids were treated for 24 h with 10 μM 7ACC2, irradiated with 6 or 9 Gy, and monitored for growth over 14 days. Neither 7ACC2 treatment alone nor radiation at 6 Gy produced significant changes in spheroid growth at day 14 (Figure 5–e). In contrast, a 9 Gy radiation dose significantly reduced spheroid size (−10.3%, *p* < 0.0001) and growth rate (−16.8%, *p* = 0.0046; Figure 5f–g). Notably, pretreatment with 7ACC2 before 9 Gy irradiation produced a far greater effect, reducing spheroid size (−40.7%, *p* < 0.0001) and growth rate (−71.1%, *p* < 0.0001). This enhanced suppression of spheroid growth following MPC inhibition is likely attributable to improved oxygenation.

### 3.6 Characterization and Cellular Uptake of Liposome-7ACC2 (Lipo-7ACC2)

Liposome assembly is influenced by lipid composition and processing parameters, such as phospholipid-to-cholesterol (PL:Chol) ratio, total flow rate (TFR), and flow rate ratio (FRR), which collectively determine the liposome size and polydispersity. Ideally, liposomes utilized for drug delivery applications should range between 50 to 200 nm, with average PDI ≤ 0.3, ensuring stability and therapeutic efficacy. We investigated the effects of phospholipid type, PL:Chol ratio, TFR, and FRR on liposome characteristics (Figures S5 and S6). DMPC formulations with a 2:1 PL:Chol ratio consistently produced liposomes ≤ 200 nm (Figure S5a) with a low PDI (0.085–0.305; Figure S6a). However, a 3:1 PL:Chol ratio destabilized DMPC liposomes, doubling PDI values (Figure S6a). DPPC liposomes showed size reduction from 275 to 176 nm (*p* < 0.0001) when FRR increased from 1:2 to 1:4 at 2:1 PL:Chol and TFR 1 mL/min, but increased significantly in terms of both size and PDI at TFR 4 mL/min (Figure S5b & S6b). DSPC and DOPC formulations exhibited less favorable trends: DSPC liposomes increased in size with a higher FRR at 3:1 PL:Chol and TFR 4 mL/min (1.8-fold, *p* = 0.0065 – Figure S3c), while DOPC liposomes showed opposite size responses depending on the PL:Chol ratio, with significant PDI increases at high TFR and FRR (Figures S3d and S4d).

Based on favorable size and PDI criteria (Table S4), DMPC and DPPC liposomes prepared at TFR 4 mL/min and FRR 1:3 were selected for 7ACC2 encapsulation. DSPC and DOPC were excluded from further studies. Drug loading had minimal impact on DPPC liposomes’ characteristics, maintaining a size <200 nm and PDI ≤ 0.3 across concentrations up to 100 μM (Figure S7a,b). DMPC liposomes remained stable up to 40 μM but increased to 257 nm at 50 μM (1.6-fold, *p* = 0.014—(Figure S7a,b)). Overall, DPPC liposomes demonstrated optimal characteristics and so were progressed for subsequent investigations.

Liposomal 7ACC2 nanoparticles (Lipo-7ACC2) were prepared by using a microfluidic (MF) approach. As a first step, empty DPPC liposomes were formulated, as described above. The resultant liposomes displayed favorable characteristics, including a mean diameter of 144 nm, low PDI (0.137), with excellent homogeneity. Following drug loading, DPPC liposomes retained sizes below 200 nm across 7ACC2 concentrations up to 100 μM (Figure 6a), with PDI values consistently <0.3 (Figure 6b). The average ζ-potential of unloaded DPPC liposomes was −29 mV, and incorporation of 7ACC2 did not significantly alter this parameter (Figure 6c).

**Figure 6:**
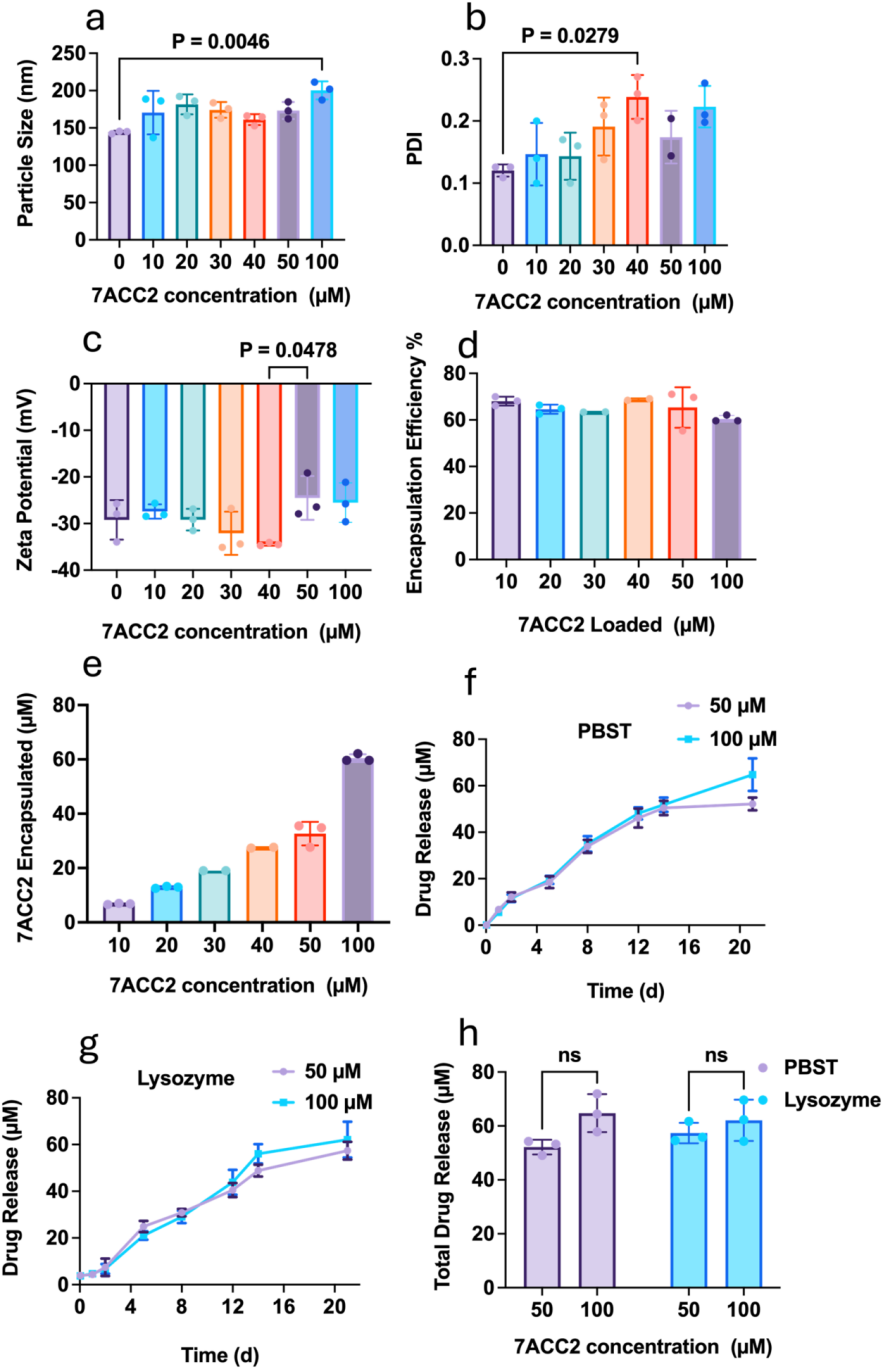
Characterization of 7ACC2-encapsulated liposomes. Dynamic light scattering (DLS) characterization of DPPC liposomes loaded with varying concentrations of 7ACC2 (0–100 μM): (a) average particle size, (b) polydispersity index (PDI), and (c) ζ-potential. (d) Encapsulation efficiency of DPPC liposomes at different 7ACC2 concentrations. (e) Quantification of the actual 7ACC2 incorporated into liposomes. (f, g) Time-dependent in vitro release profiles of 7ACC2-loaded liposomes in PBST or lysozyme-containing PBS. (h) Cumulative 7ACC2 release from liposomes after 21 days of incubation. Data represent mean ± SD from three independent experiments. Statistical differences were assessed by one-way ANOVA with Tukey’s multiple comparison test; detailed statistical values are provided.

AFM characterization demonstrated that empty liposomes generally exhibited semicircular, relatively uniform shapes (Figure S8a), although some irregularities were observed, effects likely resulting from the drying process, which can impact liposome structure and uniformity. Following 7ACC2 encapsulation, liposomes exhibited a more circular and homogeneous morphology, consistent with the DLS and PDI data (Figure S8b). This improved uniformity was caused by 7ACC2 incorporation, promoting tighter bilayer packing.

7ACC2-loaded liposome stability was next assessed by monitoring size and PDI following synthesis and under various stressed conditions. DPPC liposomes proved stable, maintaining a mean hydrodynamic size <200 nm and PDI < 0.3, across all tested drug concentrations at 37 °C (Figure S9a,b). At 4 °C, no significant change in size was observed over the four-week test period, except for the highest drug-loaded formulation (1000 μM) with a small but significant decrease by week 4 (Figure S9c). In all instances, the PDI value, irrespective of drug loading or storage duration, remained <0.3, indicating excellent stability over time (Figure S9d). Under ionic stress, both 100 and 1000 μM 7ACC2-loaded liposomes remained stable for at least 24 h in 0.15 M NaCl, with a slight size reduction from 213 to 180 nm (*p* = 0.0004) after a week of exposure (Figure S10a,b). While some of these size changes proved statistically significant, none shifted liposomal characteristics outside the desired size and PDI parameters.

MF-based preparations are known to substantially enhance drug loading compared with conventional methods, such as film hydration or extrusion. Using MF, DPPC liposomes achieved encapsulation efficiencies (EE) of 63.4–68.5% across all tested concentrations (Figure 6d), corresponding to 7ACC2 loadings from 6.8 μM to 60.5 μM at the lowest and highest drug inputs, respectively (Figure 6e).

The release profile of 7ACC2-loaded DPPC liposomes was evaluated in PBST (2% Tween 80 in PBS) and PBS supplemented with lysozyme (3 mg/mL), simulating physiologically relevant conditions. Two formulations (50 and 100 μM input concentrations) corresponding to 32 and 60 μM actual drug loadings were selected. In PBST, release was gradual and sustained, reaching 12.1 and 11.5% at day 2, and increasing to 52.2 and 64.7% by day 22 (Figure 6f). In lysozyme-containing PBS, release was delayed, remaining low at day 2 (7.45 and 6.8%), but increased significantly thereafter, reaching 57.3 and 62% by day 22 (Figure 6g). Despite differences in initial loading, the absolute cumulative release at day 22 was similar between formulations (Figure 6h). *In vitro* release data were then fitted to the Korsmeyer–Peppas equation, yielding strong correlations (*R* ^2^ = 0.90–0.95; Table S5). Notably, the *K* value was higher for 50 μM-loaded liposomes (21.6 vs 9.8–11.8 for lysozyme and PBST, respectively), suggesting faster release kinetics. However, n values for all formulations (0.623 and 0.794) indicated that drug release followed non-Fickian transport.

### 3.7 Lipo-7ACC2 Radiosensitizes Spheroid Models by Suppressing Oxygen Consumption

Given the preferential cell internalization of liposomes, we next investigated whether encapsulated 7ACC2 retained its radiosensitizing potential in both 2D monolayers and 3D spheroids. Neither empty liposomes nor Lipo-7ACC2 induced significant toxicity in PC3 or FaDu cells (Figure S11a,b). Consistent with free 7ACC2, Lipo-7ACC2 treatment sensitized FaDu cells (SER = 1.12; Figure S11c and Table S6) and produced a similar effect in PC3 cells to radiation (SER = 1.19, *p* = 0.0074; Figure S11d and Table S6).

Importantly, encapsulation of 7ACC2 in DPPC liposomes did not impair its ability to disrupt TCA cycle activity, significantly decreasing intracellular succinate levels in PC3 (−20.8%, *p* = 0.0068) and FaDu cells (−39.5%, *p* = 0.0002). Furthermore, addition of chloroquine, a recognized endosomal disruption agent, resulted in no further reduction in succinate, indicating lipo-7ACC2 was not constrained by endosomal entrapment (Figure 7a-i and ii). As with free 7ACC2, Lipo-7ACC2 markedly reduced Image-iT hypoxia fluorescence compared with untreated controls (24.38 au vs 44.03 au; Figure 7b), whereas empty liposomes had no measurable effect. Quantification of the hypoxic ratio further confirmed a significant reduction with Lipo-7ACC2 treatment alone (−19.8%, *p* < 0.0001; Figure 7c). Combining Lipo-7ACC2 with radiation did not produce additional reductions in the hypoxia.

**Figure 7:**
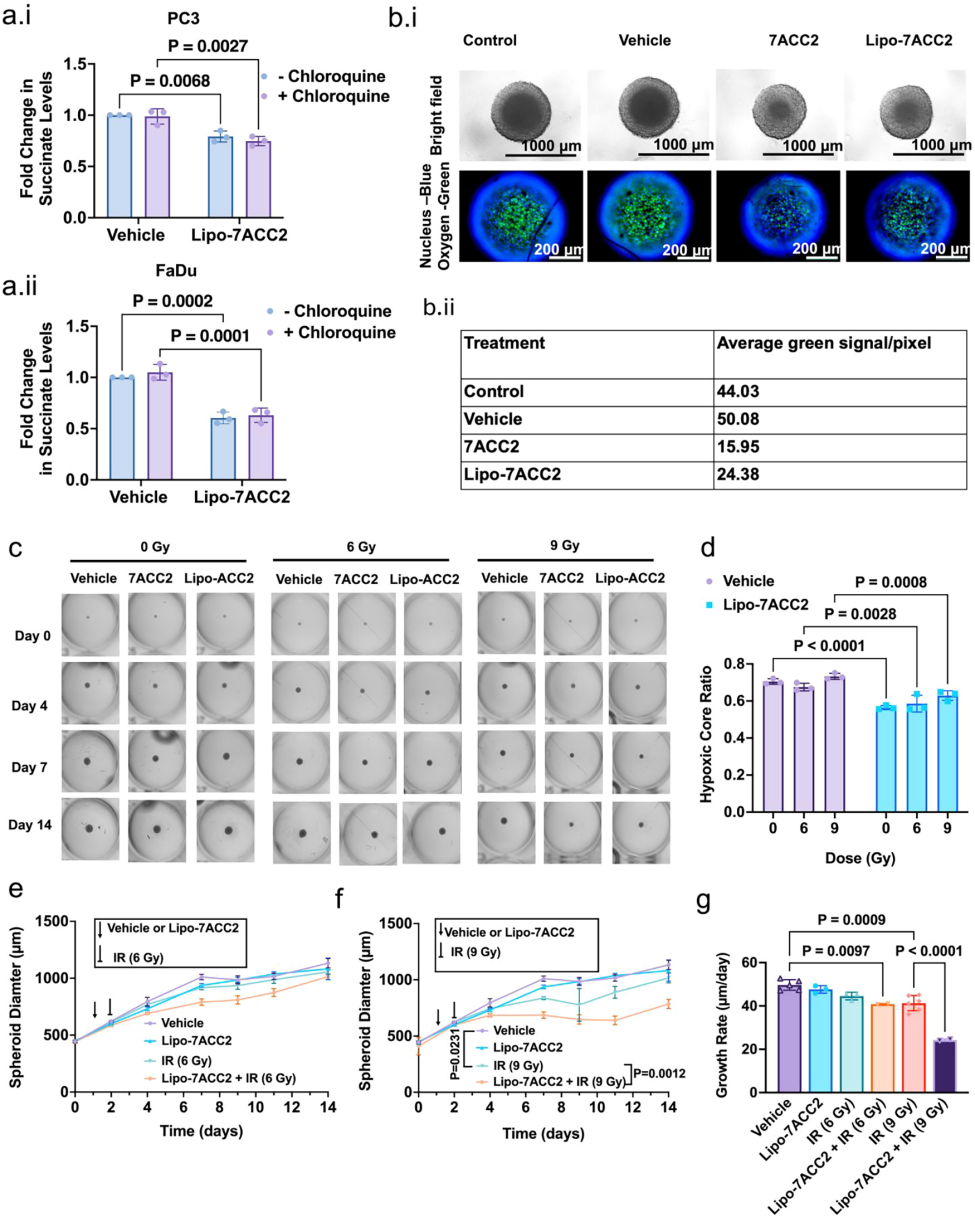
Lipo-7ACC2 enhances radiation response in 3D-spheroid models. (a) Lipo-7ACC2-mediated reduction in succinate, a metabolic intermediate of the TCA dependent on pyruvate, in (i) PCe and (ii) FaDu cells ± chloroquine, an endosomal disruption agent. (b) fluorescence and bright-field images of FaDu spheroid (∼600 μm) treated with DMSO, 7ACC2, or Lipo-7ACC2 for 24 h, stained with Image-iT green hypoxia dye and Hoechst nuclear counterstain. (b) (ii) Quantification of Image-iT green hypoxia fluorescence via ImageJ color histogram analysis. (c) Representative images of unirradiated or irradiated FaDu spheroid treated with DMSO, 7ACC2, or Lipo-7ACC2. (d) Quantitative analysis of the necrotic/hypoxic core ratio in unirradiated or irradiated spheroid. Spheroid growth delay assay (SGD): FaDu spheroids were treated with DMSO, 7ACC2, or Lipo-7ACC2 for 24 h, followed by 6 or 9 Gy radiation. Growth was monitored over 14 days (*n* ≥ 8 spheroids per condition). (e, f) SGD curves and (g) growth rate, calculated as spheroid diameter increase per day. Statistical analysis: For all other experiments, statistical differences were determined by using a one-way ANOVA with a Tukey multiple comparison test.

Finally, we tested whether alleviation of spheroid hypoxia using Lipo-7ACC2 translated to an improved radiation response. FaDu spheroids were treated with 10 μM Lipo-7ACC2 for 24 h, irradiated at 6 or 9 Gy, and monitored for growth over 14 days (Figure 7c,d). Neither Lipo-7ACC2 alone, 6 Gy irradiation, nor the combination had any significant radiation dose modifying effect. Unsurprisingly, radiation alone (9 Gy) significantly reduced spheroid growth by ∼11% (*p* < 0.0023) over vehicle alone (Figure 7f). However, combined Lipo-7ACC2 with 9 Gy radiation significantly increased spheroid growth delay by 21.3% (*p* = 0.0012), reducing the growth rate from 41 μm/day to 23 μm/day (Figure 7f,g).

## 4 Discussion

Dysregulated lactate metabolism, observed in prostate and head-and-neck cancers,^3,38^ drives tumor progression, microenvironmental remodeling, therapeutic resistance, and poor patient outcomes.^38^ Targeting lactate metabolism could serve as a promising strategy to enhance conventional therapies with MCT1/4 and MPC identified as potential regulators of radiosensitivity.

MCTs are frequently overexpressed in many cancers, including renal, breast, cervical, head and neck, and prostate tumors.^3,14,39,40^ MPC imports pyruvate into the mitochondrial matrix for oxidation, and unlike MCT, downregulation is linked to poor treatment outcomes because of a tumor cell shift to glycolytic reliance.^41^ In our study, PC3 and FaDu cells expressed both MCT1/4 and MPC, with higher MCT1 expression in FaDu cells, consistent with prior reports,^19^ with PC3 cell MPC expression decreasing under hypoxia, confirming oxygen-sensitive regulation.^42^

Extracellular acidification profoundly impacts tumor progression and therapy response.^43^ We observed that MCT1 inhibition via AZD3965 increased the pHe in PC3 and FaDu cells, while MCT4 inhibition had modest effects. Conversely, MPC inhibition with 7ACC2 induced extracellular acidification, consistent with studies showing that blocking lactate efflux results in accumulated intracellular lactate, inhibiting glycolysis, and reactivating mitochondrial metabolism helping sustain survival.^44^ Similarly, Buyse et al. reported that MPC inhibition enhances glucose consumption, lactate secretion, and acidification,^45^ though effects were suppressed *in vivo* due to tumor heterogeneity.

Inhibiting lactate metabolism has recently emerged as a promising antitumor strategy to enhance conventional therapies,^16^ with current approaches targeting lactate either directly or in combination with other treatments. Blocking MCTs disrupts lactate exchange between cells, suppressing proliferation, invasion, and metastasis. In this study, dual MCT1/4 inhibition induced significant cell death under hypoxia. Notably, MCT1 inhibition alone was ineffective, consistent with reports that MCT4 compensates for MCT1 loss,^21^ driving resistance to AZD3965. By comparison, MCT4 ablation alone was sufficient to kill PC3 cells, in line with reports showing that hypoxia-induced HIF-1α promoted expression of MCT4 is an essential metabolic adaptation, particularly in breast cancer.^12,46^

Tumor cells continuously sense nutrient and oxygen availability in the tumor microenvironment, adjusting metabolism accordingly. While glucose is the primary fuel, tumor cells can reprogram to use substrates such as glutamine and lactate.^47^ We found that under hypoxia and glucose starvation, blocking extracellular lactate uptake with MCT1 inhibition suppressed cancer growth when lactate was the sole nutrient source. MCT1 is reported to exhibit increased affinity for lactate following glucose deprivation under hypoxia, with p53-deficient tumors elevating MCT1 to promote lactate import, fuelling mitochondrial respiration.^48^ Directly linked to this, 7ACC2-mediated MPC inhibition blocks mitochondrial import of pyruvate, inhibiting respiration and tumor cell growth. These findings underscore that lactate uptake under hypoxia provides an alternative fuel that sustains survival during glucose deprivation. However, it is interesting to note that under glucose-rich conditions, neither MCT1 nor MPC inhibition alone triggered significant cell death. To achieve an antitumor impact in nutrient-rich conditions, combined MCT1 and MPC inhibition was required, with MCT4 augmenting the response, potentially indicating that MCT4-mediated lactate export mediates metabolic adaption when glucose is plentiful.

Radiotherapy remains a curative treatment for many cancers, including head-and-neck and prostate tumors.^49,50^ However, elevated glycolysis and lactate accumulation drive radioresistance by promoting antioxidant defenses and an immunosuppressive microenvironment.^51^ While MCT1/4, MPC, and LDHA have been linked to radiosensitivity,^52^ our data show that MCT1 inhibition alone failed to enhance radiosensitivity in PC3 or FaDu cells, likely due to MCT4 compensation. In contrast, MPC inhibition with 7ACC2 was a key driver of radiation sensitivity (Figure 3). Seahorse analysis demonstrated that 7ACC2 reduced OCR by 20% in PC3 and 35% in FaDu cells, consistent with reports that describe OCR reductions alleviating hypoxia and enhancing radiation sensitivity.^53,54^ MPC inhibition also reduced intracellular succinate, a TCA cycle intermediatory that inversely impacts the HIF-1α hydroxylase activity of prolyl hydroxylase domain (PHD) enzymes, marking HIF-1α for degradation even under hypoxic stress.^55^ In 3D-FaDu spheroids, MPC inhibition decreased hypoxia, confirming the radiosensitizing potential of 7ACC2 in more physiologically relevant models.

Despite its efficacy, 7ACC2 is limited by poor water solubility and a short half-life.^28^ Liposomal encapsulation offers a potential solution, improving stability and delivery, while providing sustained release. Using an MF approach, we encapsulated 7ACC2 in DPPC liposomes (<200 nm) with low polydispersity and 63.4–68.5% encapsulation efficiency. Drug release kinetics are critical for predicting PK/PD, optimizing dosing schedules, and evaluating delivery.^56^ Both 50 and 100 μM liposome formulations exhibited sustained 7ACC2 release over 21 days, with 50 μM liposomes showing a 2-fold higher release constant (*K*) and non-Fickian diffusion (*n* = 0.45–0.89), indicating boundary-mediated passive diffusion.^57,58^ *In vivo*, 7ACC2 has a short half-life (4.5 h at 3 mg/kg),^28^ yet sensitizes SiHa xenograft tumors to radiation using a daily dosing regimen combined with fractionated radiotherapy.^26^ These prior studies, coupled with the data presented within this work, support liposomal encapsulation of 7ACC2 as a strategy to mitigate the impact of its short half-life while enabling prolonged release. While a hypothesis at the current state of development, this approach could reduce the need for daily injections, lowering treatment-related burden and improving treatment outcomes. However, further *in vivo* studies are necessary to confirm these advantages and fully characterize the pharmacokinetic and therapeutic benefits of this liposome formulation.

Importantly, Lipo-7ACC2 retained its ability to inhibit TCA cycle activity and radiosensitize tumor cells in both 2D and 3D models (Figure 7), confirming drug release at effective concentrations. In FaDu spheroids, Lipo-7ACC2 reduced spheroid size and necrotic score while lowering Image-iT hypoxia fluorescence, demonstrating effective diffusion, improved oxygenation, and reversal of hypoxia-driven radioresistance.^59^ Combined with radiotherapy, Lipo-7ACC2 achieved maximal reduction in spheroid growth and size, outlining the potential for continued development.

## 5 Conclusion

In conclusion, our study highlights the role of lactate in fueling mitochondrial respiration and sustaining tumor growth under hypoxia. Inhibiting MPC with 7ACC2 disrupts this metabolic symbiosis, promotes reoxygenation, and sensitizes tumors to radiation. Importantly, liposomal encapsulation preserves the radiosensitizing effects of 7ACC2 in both 2D and 3D tumor models while overcoming its pharmacological limitations, offering a simple and effective strategy to enhance metabolic radiosensitization and combat hypoxia-driven radioresistance.

## Supplementary Material



## Notes

Schematic illustration of 7ACC2-encapsulated liposome via microfluidic approach (Figure S1); the effect of inhibitors of lactate and pyruvate transport on extracellular pH (pHe) (Figure S2); clonogenic radiation curve for PC3 and FaDu cells under both hypoxia and normoxia (Figure S3); SF_0Gy_ value of FaDu, and PC3 cells after treatment with AZD3965, 7ACC2, combined AZD3965 and 7ACC2, and the triple treatment of AZD3965, 7ACC2, and syrosingopine (Figure S4); DLS size characterization of liposomes prepared with different parameters, including phospholipid: cholesterol (PL:Chol) ratio, total flow rate (TFR) and flow rate ratio (FRR) (Figure S5); PDI value of liposomes prepared with different parameters, including phospholipid: cholesterol (PL:Chol) ratio, total flow rate (TFR) and flow rate ratio (FRR) (Figure S6); characterization of 7ACC2-loaded liposomes (DMPC, DPPC) using different concentration of 7ACC2 (Figure S7); AFM images of empty and 7ACC2-loaded DPPC liposomes; stability of 7ACC2-loaded DPPC liposomes under different storage temperatures (Figure S9); stability of 7ACC2-loaded DPPC liposomes under salt (0.15 M) induced stress (Figure S10); detailed characterization of Lipo-7ACC2 in PC3 and FaDu cells (Almarblue assay and 2D clonogenic assay) (Figure S11); detailed information on buffer-free medium composition (Table S1); oxygen enhancement ratio (OER) values for PC3 and FaDu cells (Table S2); sensitizer enhancement ratio (SER) and dose enhancement factors (DEF) of PC3 and FaDu cells after treatment with AZD3965, 7ACC2, or the combination with syrosingopine under both normoxia (21% O_2_) and hypoxia (0.5% O_2_) (Table S3); size and PDI values for each lipid (DMPC, DPPC, DSPC, and DOPC) under optimal microfluidic parameters (Table S4); parameters *k*, *n*, and r2 of nonlinear data fitting Korsmeyer–Peppas model for 50 and 100 μM 7ACC2-loaded DPPC liposome (Table S5); and sensitizer enhancement ratio (SER) and dose enhancement factor (DEF) of PC3 and FaDu cells after treatment with 7ACC2 or Lipo-7ACC2 (Table S6) (PDF)
