# Supplementary material for: Metabolic Radiosensitization by Targeting Lactate Metabolism with Microfluidic Liposomal Nanocarriers
Source: ACS Biomater Sci Eng. 2026 Jan 28;12(2):1018–31. doi: 10.1021/acsbiomaterials.5c02175 (PMC12892243; doi:10.1021/acsbiomaterials.5c02175)
Supplement: Supplementary file 1 [file ab5c02175_si_001.pdf]

Metabolic Radiosensitisation by Targeting Lactate Metabolism with Microfluidic Liposomal Nanocarriers

*Meabh Doherty [a], Jie Feng [a], Tongchuan Wang [a], Cancan Yin [a], Niall M. Byrne [a], Sarah Chambers [a], Rayhanul Islam [a], Dimitrios A Lamprou [a], Jonathan A. Coulter [a] **

a. School of Pharmacy, Queens University Belfast, Lisburn Road, Belfast BT9 7BL, UK

*Corresponding author: j.coulter@qub.ac.uk

Extracellular pH (pHe) measurement

For the determination of pHe, Cells were seeded into 24-well plates and left to adhere overnight at 37 ˚C. Subsequently cell media was replaced with fresh buffer-free (Table S1) media containing AZD3965 (1 μM), syrosingopine (10 μM), 7ACC2 (μM) or in combination and then incubated for another 72h. Then 500 μL of cell media was taken out for measurement using pH meter (Hanna instruments).

Alamar Blue Cell Viability Assay

The therapeutic potential of targeting lactate metabolism was investigated using Alamarblue assay. Briefly, PC3 and FaDu cell were plated in 96-well plates at a density of 10^4 cells/well and allow to adhere overnight. After that, cells were then treated with fresh medium containing MCT1 inhibitor AZD3965(1 μM), MCT4 inhibitor syrosingopine (10 μM) or MPC inhibitor 7ACC2 (10 μM) or in combination and incubate for another 24h. For conditioned assay, after cell adhesion and washing, cells were then incubated with either complete medium (media containing glucose (10mM) or glucose-free medium (supplemented with lactate (10 mM) and then exposed to AZD3965 (1 μM), syrosingopine (10 μM), 7ACC2 (10 μM). After 24 h, cells were then washed with PBS and incubated with 10% Alamar blue in complete media for another 4h, after which 100 μL of cell media was transferred to black 96-well plate, and the fluorescence level were then measured at 570 nm excitation and 585 nm emission using the Flurostar plate reader. Cell viability was assessed by comparing the fluorescence level of each treatment group to that of the vehicle.

Stabilities study

Systematic study on size distribution and stability was carried out in 7ACC2 loaded liposomes with different loading concentration (200-1000 μM) under different storage temperatures of 37°C (1 week) and 4°C (up to 4 weeks), respectively. Three independent replicates of each formulation were prepared, and the stability of 7ACC2 loaded liposomes were examined by measuring the size and PDI after storage of formulation at 37°C for 1 week, with additional stressing testing conducted under 150 mM NaCl (physiological ionic strength).

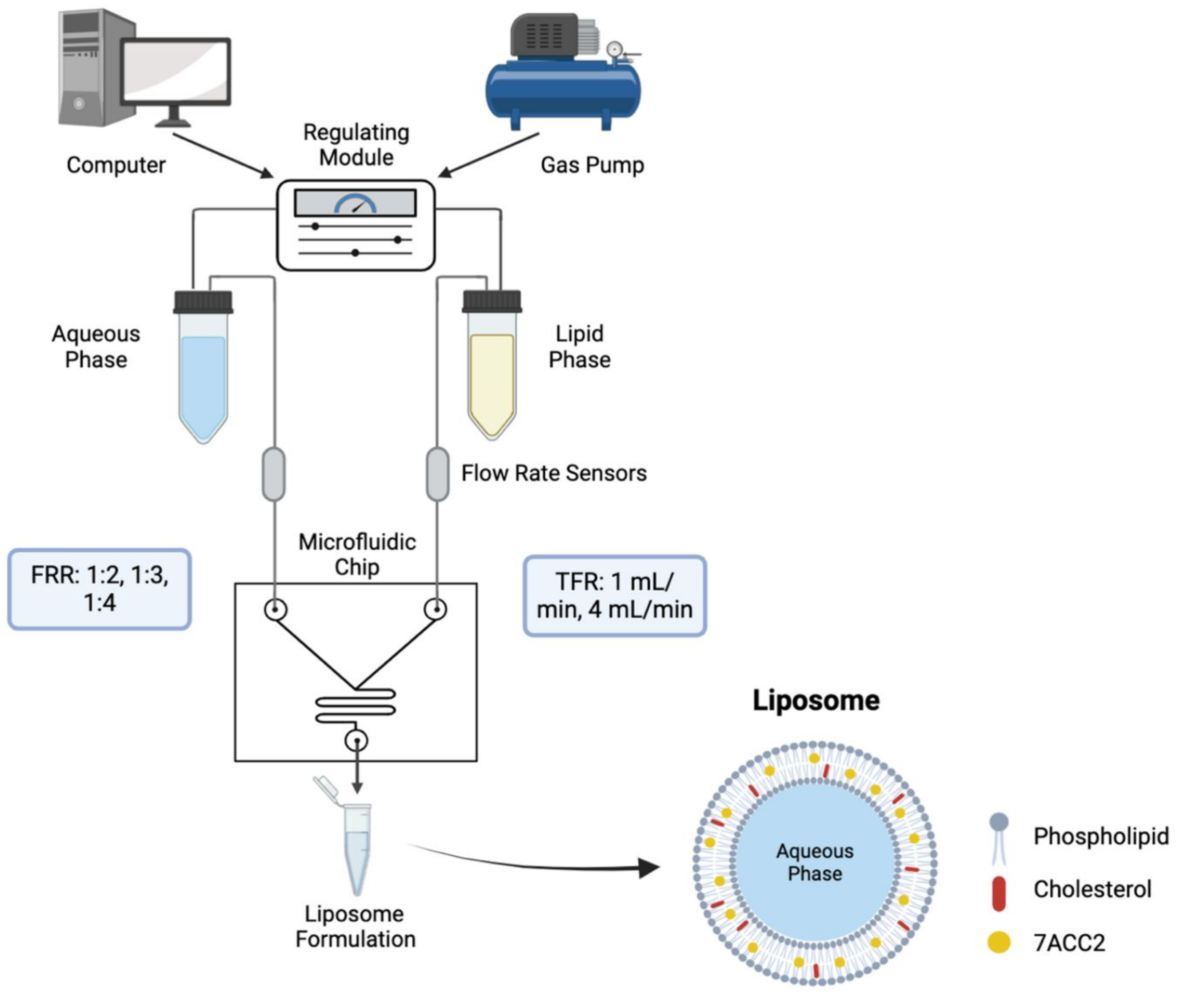

Figure S1 Schematic illustration of preparation of 7ACC2 encapsulated liposome via microfluidics using Y shaped chip (figure created using Biorender.com). The lipid phase (containing 7ACC2) and aqueous phase were injected into Y-shaped microfluidic chip with different flow rate ratio (lipid: aqueous ratio of 1:2, 1:3, and 1:4) and total flow rate (1 and 4 mL/min). 7ACC2 was loaded into the lipid phase and encapsulated within lipid bilayer.

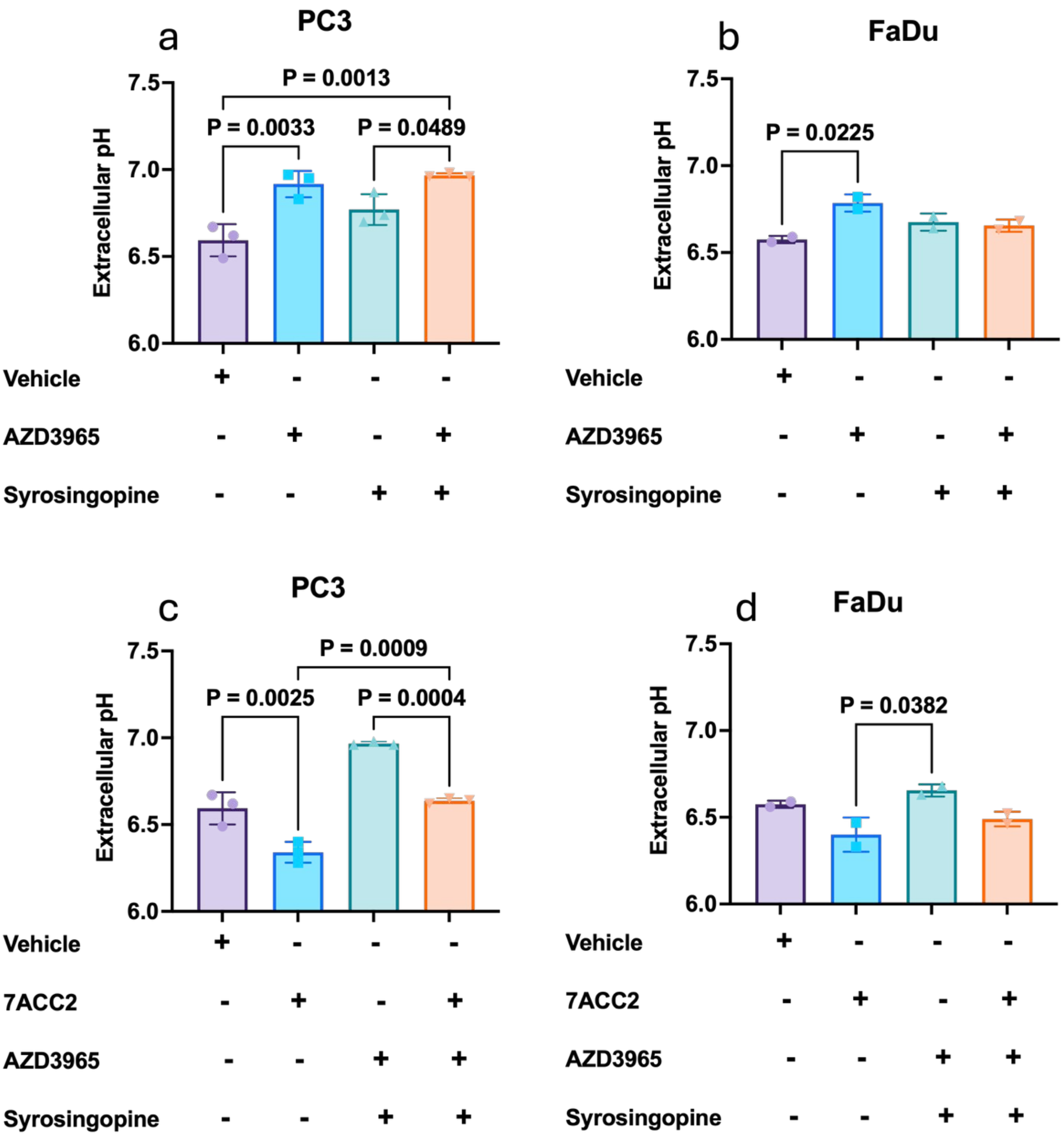

Figure S2. Inhibitors of lactate and pyruvate transport differentially impact extracellular pH (pHe). pHe was measured in PC3 **(a, c)** and FaDu cells **(b, d)** after 72 h treatment with AZD3965 (1 µM), syrosingopine (10 µM), 7ACC2 (10 µM), or combinations. Data are presented as mean ± SD from three independent replicates. Statistical significance was assessed using one-way ANOVA followed by Tukey's multiple comparison test.

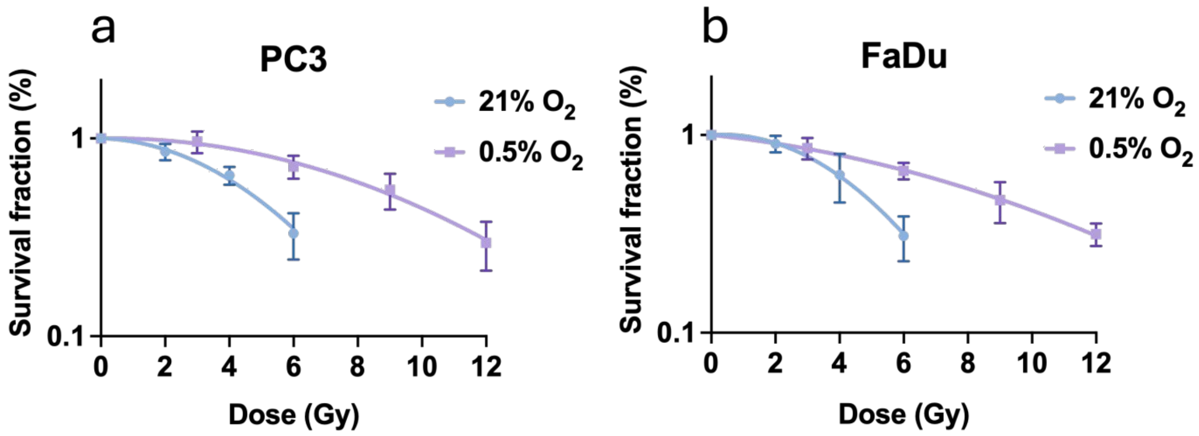

Figure S3 Clonogenic radiation survival curve for PC3 (a) and FaDu (b) cells under both normoxia and hypoxia. Data are derived from three independent replicates and reported as mean±SD.

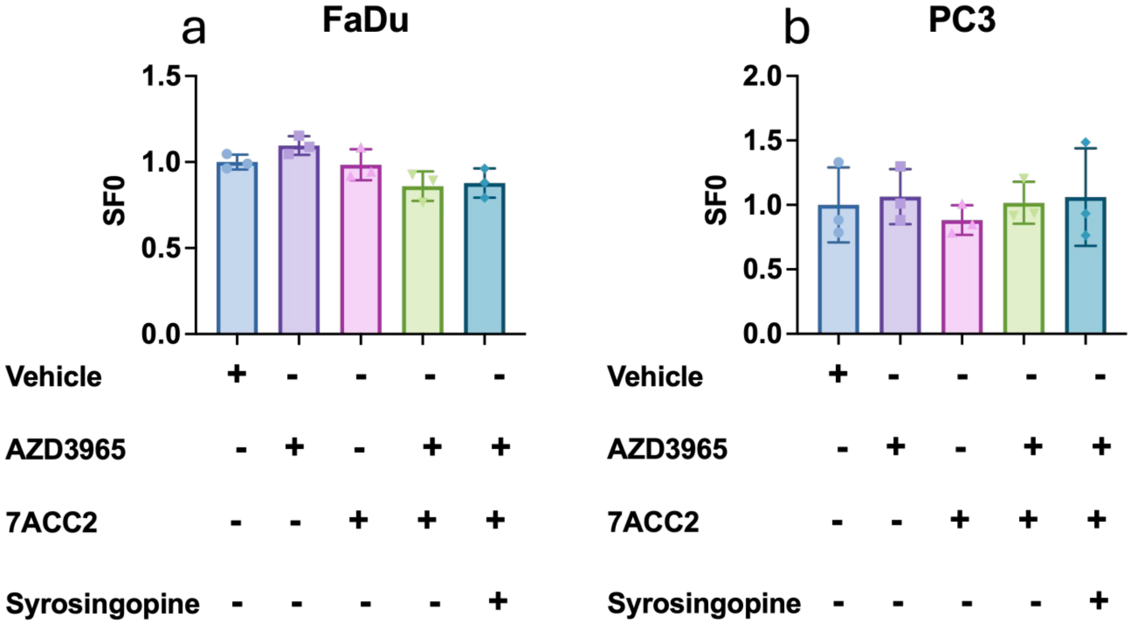

Figure S4. SF_{0Gy} value of FaDu, and PC3 cells after treatment with AZD3965 (1 μM), 7ACC2 (10 μM), or the combined treatment of AZD3965 (1 μM) and 7ACC2 (10 μM), or triple treatment with AZD3965, 7ACC2, and syrosingopine (10 μM)

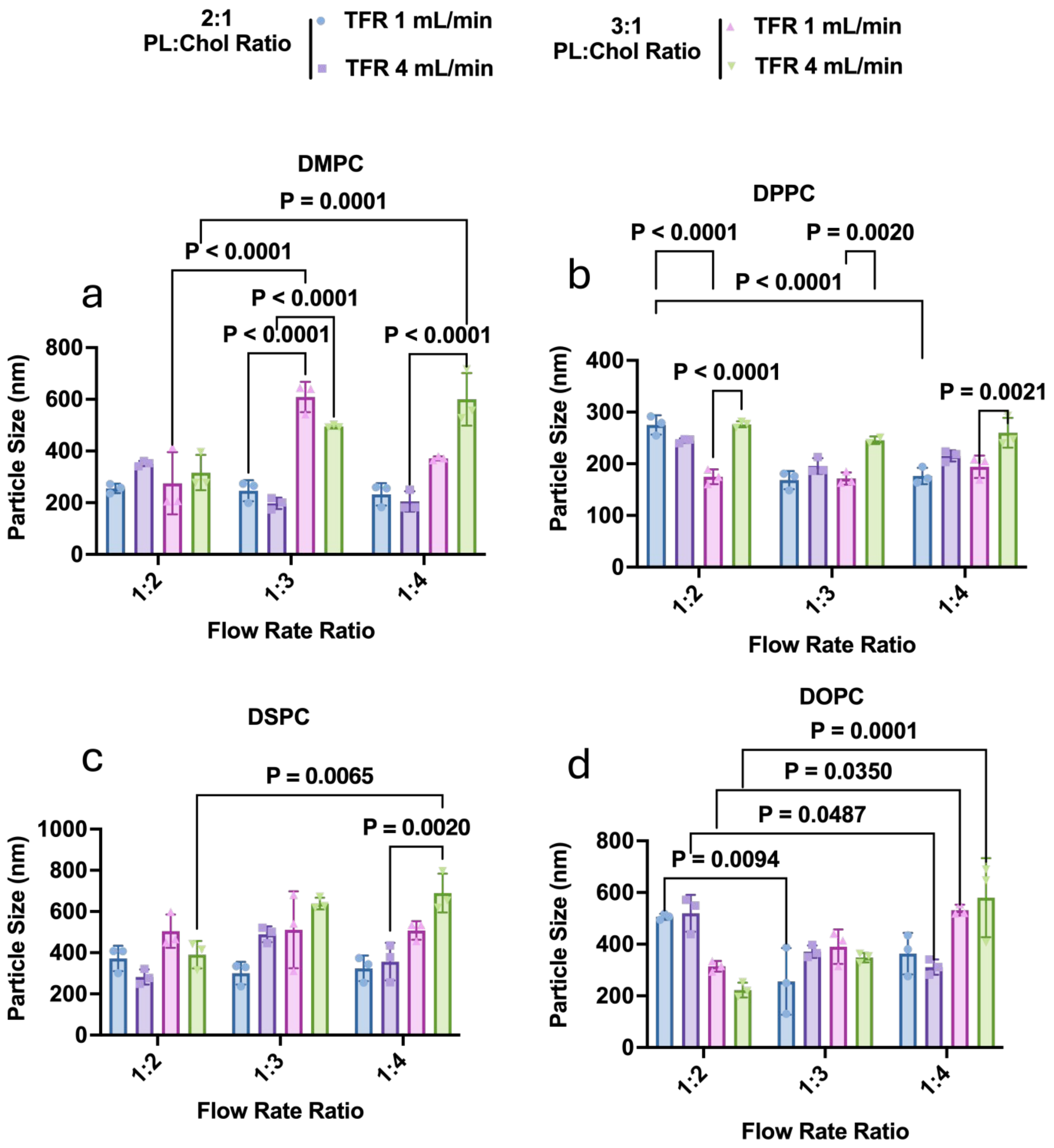

Figure S5 DLS size characterization of liposomes prepared with different parameters, including phospholipid: cholesterol (PL: Chol) ratio, total flow rate (TFR) and flow rate ratio (FRR). (a) DMPC liposome, (b) DPPC liposome, (c) DSPC liposome, (d) DOPC liposome. Data are derived from three independent replicates. All value reported as mean±SD. Statistical differences determined using two-way ANOVA with a Tukey multiple comparison test.

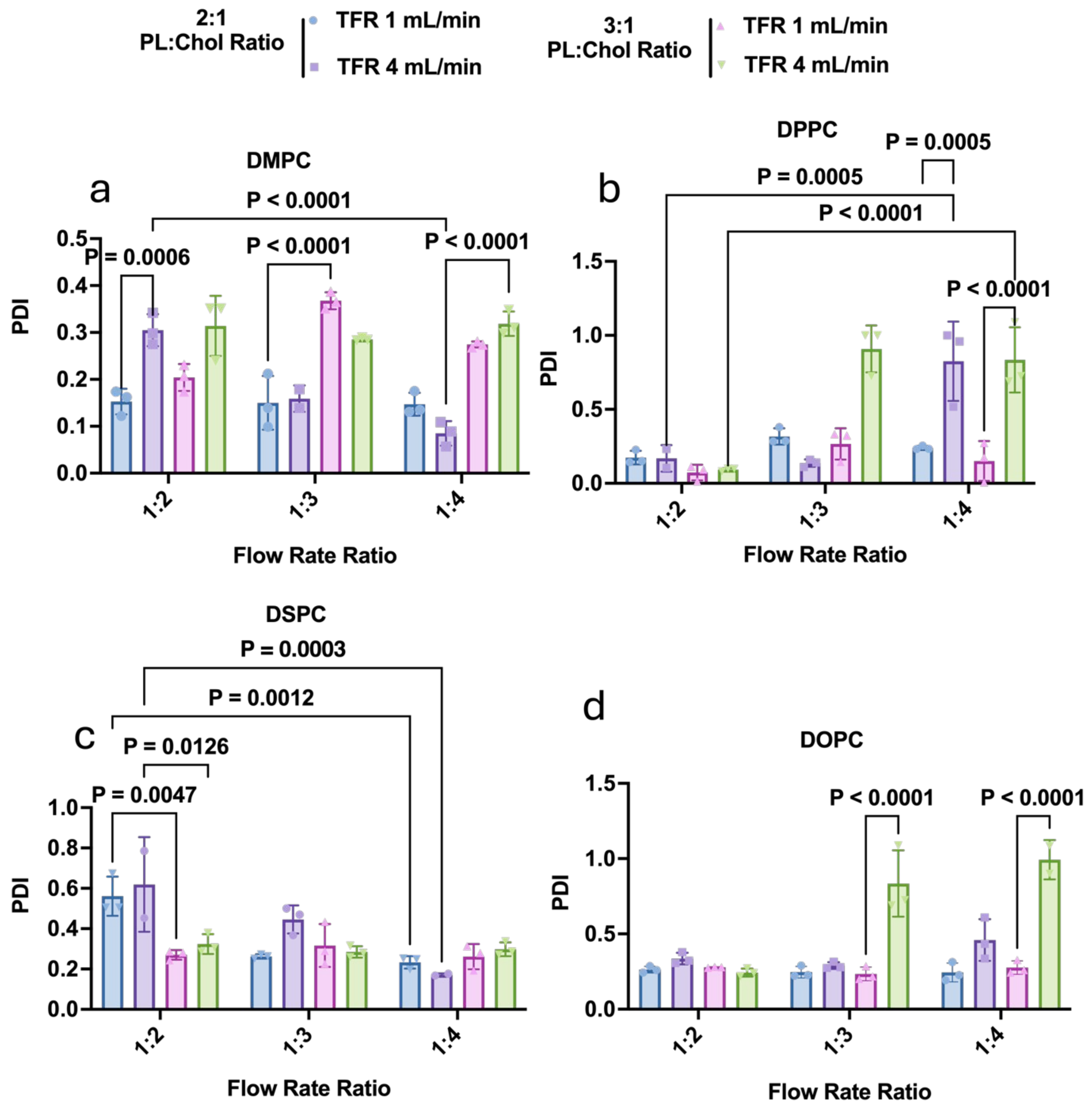

Figure S6 PDI value of liposomes prepared with different parameters, including phospholipid: cholesterol (PL: Chol) ratio, total flow rate (TFR) and flow rate ratio (FRR). (a) DMPC liposome, (b) DPPC liposome, (c) DSPC liposome, (d) DOPC liposome. Data are derived from three independent replicates. All value reported as mean±SD. Statistical differences determined using two-way ANOVA with a Tukey multiple comparison test

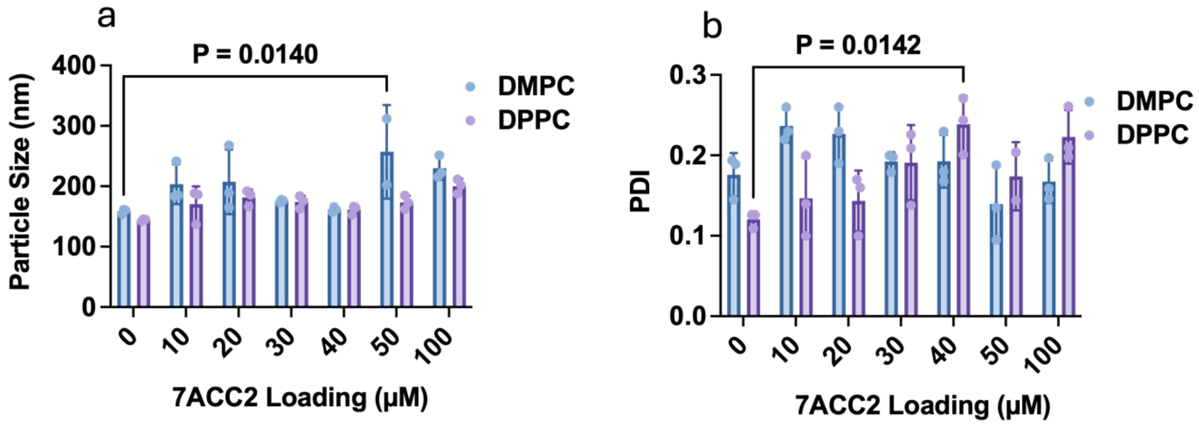

Figure S7 DLS characterization of DMPC and DPPC liposome prepared with different concentration of 7ACC2. (a) average particle size of 7ACC2 loaded DMPC and DPPC liposomes, (b) average PDI value for 7ACC2 loaded 7ACC2 loaded DMPC and DPPC liposomes. Data are derived from three independent replicates. All value reported as mean±SD. Statistical differences determined using two-way ANOVA with a Tukey multiple comparison test

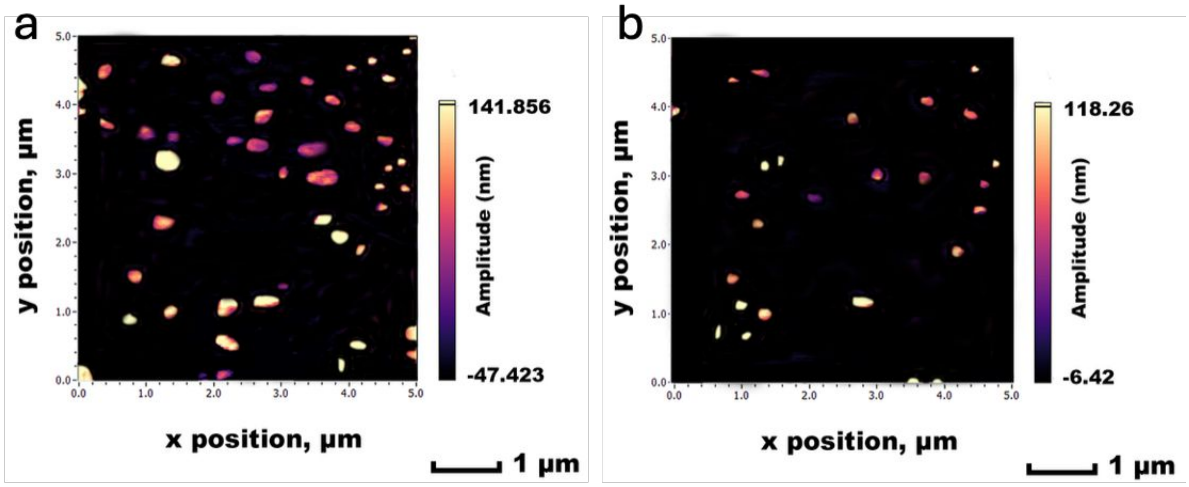

Figure S8 AFM images of empty and 7ACC2 loaded DPPC liposomes using TFR 4 mL/min and FRR of 1:3, (a) empty DPPC liposomes; (b) 7ACC2 loaded liposomes

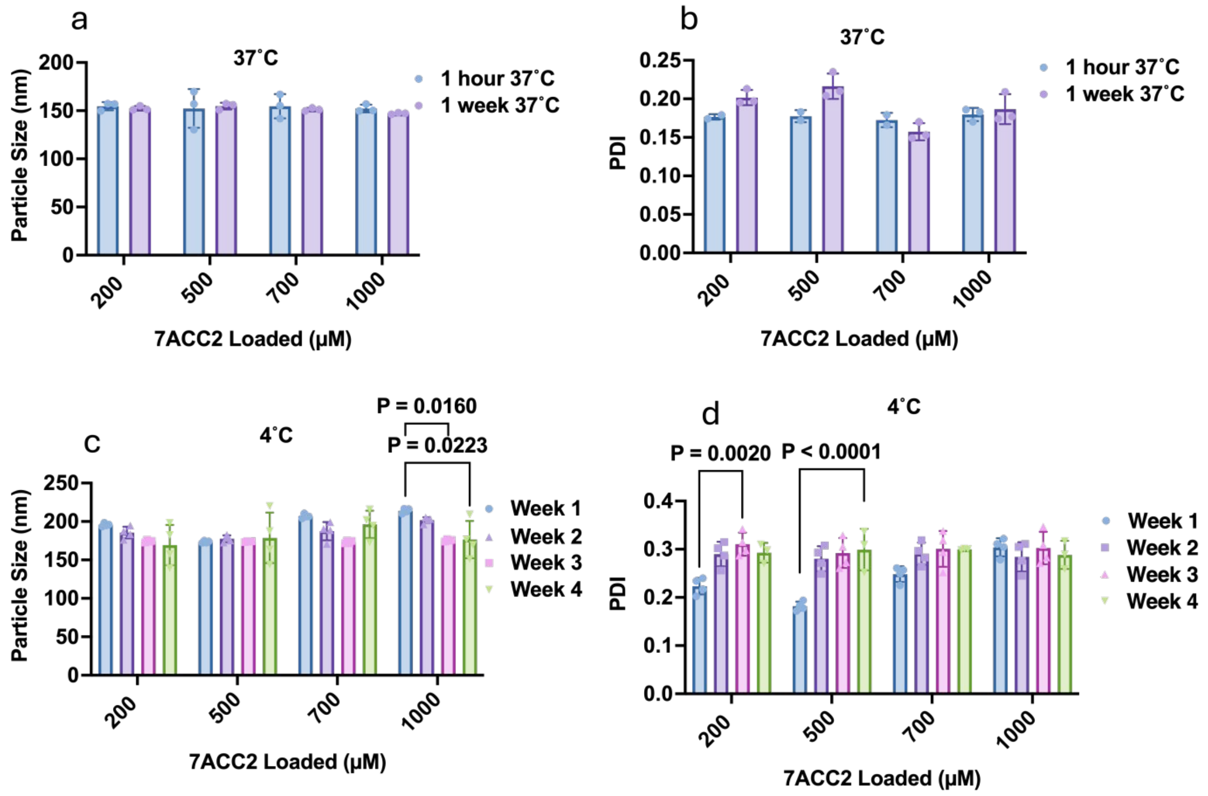

Figure S9 Stability of 7ACC2 loaded DPPC liposome under different storage temperatures. Size (a&c) and PDI (b&d) values of 200-1000 µM 7ACC2 loaded DPPC liposome after 1 week storage at 37°C, or 4 weeks storage at 4°C. Data derived from three independent experiments and plotted as mean ± SD.

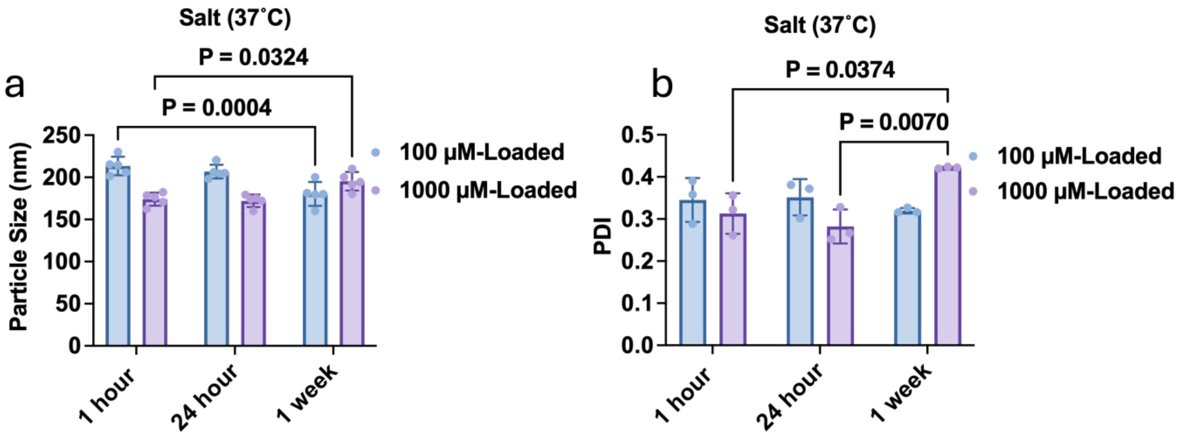

Figure S10 Stability of 7ACC2 loaded DPPC liposome under salt conditions (0.15M). Size (a) and PDI (b) values of 100 µM and 1000 µM 7ACC2 loaded DPPC liposome up to 1 week

post incubation in 0.15 M NaCl at 37°C. Data derived from three independent experiments and plotted as mean ± SD.

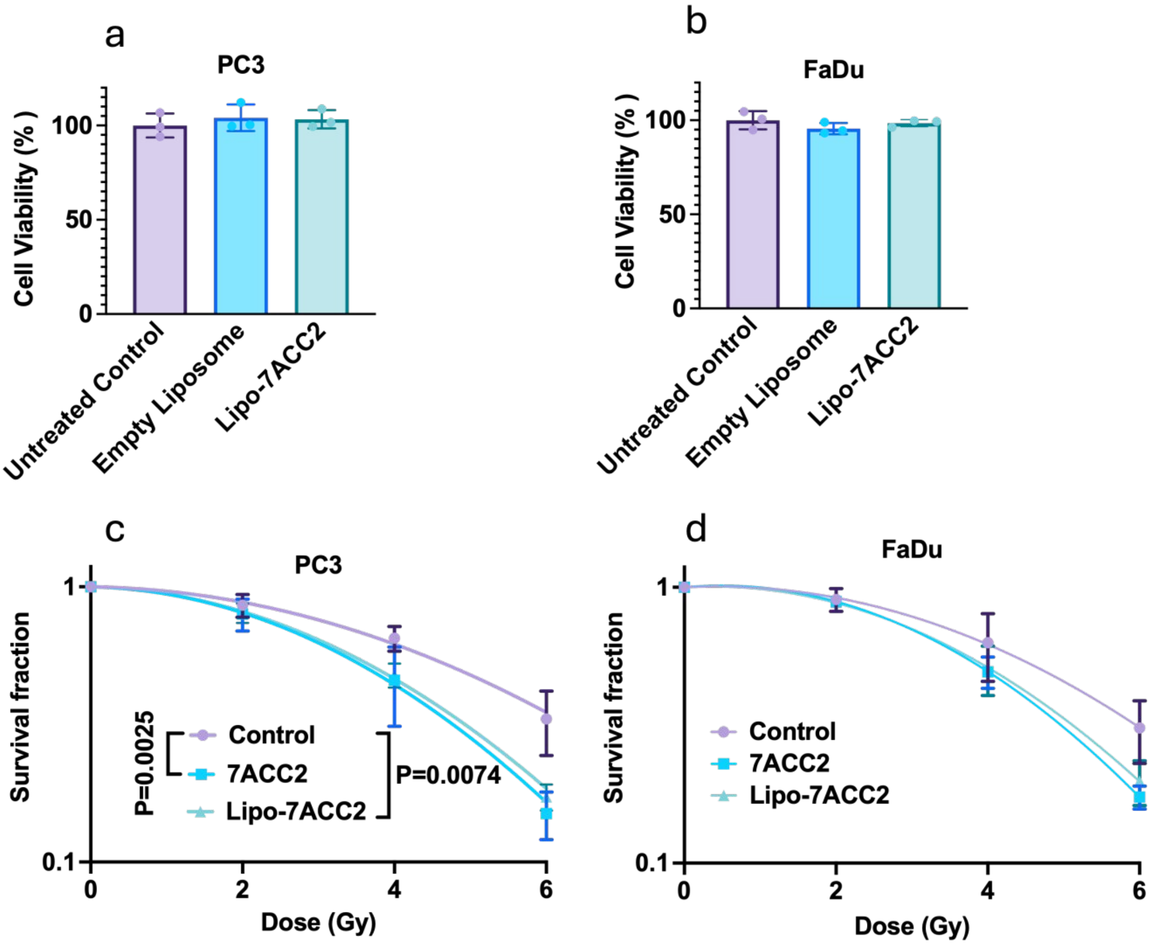

Figure S11 Lipo-7ACC2 enhances radiation response in 2D cell models. Direct cytotoxicity of (a) empty-liposome vehicle control and (b) Lipo-7ACC2 in PC3 and FaDu cells. Clonogenic radiation curves for (c) PC3 and (d) FaDu cells treated with 7ACC2 or Lipo-7ACC2 for 24 h. For clonogenic assays a two-way ANOVA with a Tukey multiple comparison test was used to determine differences between survival assay groups. For succinate assay, statistical differences were determined using statistical significance was determined using a two-tailed Student’s t-test.

Table S1. Buffer-free medium composition (100 ml)

Buffer-free medium	Volume (mL)
Minium Essential Medium Egale 10X (sigma)	81
L-Glutamine (sigma)	0.29
Water	9
FBS	10

Table S2. Oxygen Enhancement Ratio (OER) of PC3 and FaDu cells under hypoxia (0.5% O_2). OER is defined as a ratio of the radiation doses required under two differing oxygen tensions to achieve the same biological effect. In the current work this was defined as normoxia (21% O_2 verses hypoxia 0.5% O_2) reducing surviving fraction (SF) to 0.5 and 0.1, i.e. 50% and 90% cell kill. OER data was extrapolated from the clonogenic survival data.

**Cell type**	**OER_{50}**	**OER10**
PC3	1.91	1.79
FaDu	1.81	N/A

Table S3. Sensitiser enhancement ratio (SER) and dose enhancement factor (DEF) of PC3 and FaDu cells after treatment with AZD3965, 7ACC or the combination with syrosingopine under both normoxia (21% O_2) and hypoxia. SER represents area under curve versus radiation only whilst DEF value demonstrates radiation enhancement at a single dose point versus radiation only. α and β component of linear quadratic has also been presented.

**Oxygen level**	**Cell type**	**treatment**	**SER**	**α**	**β**	**α/β**	**DEF$_{3Gy}$**	**DEF$_{7.2Gy}$**
21%	PC3	Control	N/A	0.0093	0.028	0.33	N/A	N/A
		AZD3965	1.04	0.0198	0.0295	0.67	1.05	1.2
		7ACC2	1.19	0.0126	0.048	0.263	1.2	2.9
		AZD3965+7ACC2	1.21	0.024	0.048	0.5	1.25	3.2
		AZD3965+7ACC2+SSP	1.26	0.0476	0.0464	1.03	1.32	3.5
	FaDu	Control	N/A	-0.0257	0.0364	-0.706	N/A	N/A
		AZD3965	1.09	-0.0205	0.0461	-0.444	1.11	1.71
		7ACC2	1.11	-0.0578	0.0589	-0.981	1.11	2.48
		AZD3965+7ACC2	1.19	0.0212	0.0458	0.462	1.25	2.29
		AZD3965+7ACC2+SSP	1.27	0.0494	0.0458	1.08	1.36	2.82
0.5%	PC3	Control	N/A	-0.00576	0.00870	-0.662	N/A	N/A
		AZD3965	1.07	0.000292	0.0098	0.03	1.03	1.11
		7ACC2	1.2	0.00226	0.0102	2.21	1.10	1.32
		AZD3965+7ACC2	1.14	0.0138	0.0099	1.39	1.07	1.23
		AZD3965+7ACC2+SSP	1.24	0.0524	0.0071	7.38	1.17	1.40
	FaDu	Control	N/A	0.0387	0.0049	7.88	N/A	N/A
		AZD3965	1.06	0.0562	0.0043	12.97	1.05	1.10
		7ACC2	1.11	0.0622	0.0047	13.33	1.07	1.17
		AZD3965+7ACC2	1.10	0.0571	0.0050	11.31	1.06	1.15
		AZD3965+7ACC2+SSP	1.14	0.0401	0.0085	4.71	1.03	1.22

Table S4. Size and PDI values for each lipid under optimal microfluidic parameters.

Lipid	PL: Chol ratio	TFR (mL/min)	FRR	Size (nm)	PDI
DMPC	2:1	4	1:3	197	0.159
DPPC	2:1	4	1:3	195	0.137
DSPC	2:1	4	1:4	356	0.172
DOPC	3:1	4	1:2	222	0.234

Table S5. Non-linear data fitting Korsmeyer-Peppas model for 50 μM and 100 μM 7ACC2 loaded DPPC liposome.

**7ACC concentration (μM)**	**PBST**		**Lysozyme**	
	**50**	**100**	**50**	**100**
**K**	26.7	11.8	21.6	9.8
**n**	0.623	0.738	0.704	0.794
**r2**	0.979	0.995	0.992	0.988

Table S6 Sensitiser enhancement ratio (SER) and dose enhancement factor (DEF) of PC3 and FaDu cells after treatment with 7ACC2 or Lipo-7ACC2. SER represents area under curve versus radiation only whilst DEF value demonstrates radiation enhancement at a single dose point versus radiation only. α and β component of linear quadratic has also been presented.

**Cell type**	**treatment**	**SER**	α	β	α/β	**DEF_{2Gy}**	**DEF_{4Gy}**
PC3	Control	N/A	0.0093	0.02764	0.34	N/A	N/A
	7ACC2	1.22	0.0126	0.048	0.26	1.21	2.94
	Lipo-7ACC2	1.19	0.0147	0.044	0.33	1.18	2.48
FaDu	Control	N/A	-0.0257	0.0363	-0.71	N/A	N/A
	7ACC2	1.13	-0.0578	0.0584	-0.99	1.11	2.49
	Lipo-7ACC2	1.11	-0.0417	0.0524	-0.80	1.10	2.04
